# Toll-like receptor-guided therapeutic intervention of human cancers: molecular and immunological perspectives

**DOI:** 10.3389/fimmu.2023.1244345

**Published:** 2023-09-26

**Authors:** Suprabhat Mukherjee, Ritwik Patra, Payam Behzadi, Andrea Masotti, Alessandro Paolini, Meysam Sarshar

**Affiliations:** ^1^ Integrative Biochemistry & Immunology Laboratory, Department of Animal Science, Kazi Nazrul University, Asansol, West Bengal, India; ^2^ Department of Microbiology, Shahr-e-Qods Branch, Islamic Azad University, Tehran, Iran; ^3^ Research Laboratories, Bambino Gesù Children’s Hospital-IRCCS, Rome, Italy

**Keywords:** toll-like receptors (TLRs), human cancers, therapeutic interventions, immunotherapy, chemotherapy, agonists, antagonists

## Abstract

Toll-like receptors (TLRs) serve as the body’s first line of defense, recognizing both pathogen-expressed molecules and host-derived molecules released from damaged or dying cells. The wide distribution of different cell types, ranging from epithelial to immune cells, highlights the crucial roles of TLRs in linking innate and adaptive immunity. Upon stimulation, TLRs binding mediates the expression of several adapter proteins and downstream kinases, that lead to the induction of several other signaling molecules such as key pro-inflammatory mediators. Indeed, extraordinary progress in immunobiological research has suggested that TLRs could represent promising targets for the therapeutic intervention of inflammation-associated diseases, autoimmune diseases, microbial infections as well as human cancers. So far, for the prevention and possible treatment of inflammatory diseases, various TLR antagonists/inhibitors have shown to be efficacious at several stages from pre-clinical evaluation to clinical trials. Therefore, the fascinating role of TLRs in modulating the human immune responses at innate as well as adaptive levels directed the scientists to opt for these immune sensor proteins as suitable targets for developing chemotherapeutics and immunotherapeutics against cancer. Hitherto, several TLR-targeting small molecules (e.g., Pam3CSK4, Poly (I:C), Poly (A:U)), chemical compounds, phytocompounds (e.g., Curcumin), peptides, and antibodies have been found to confer protection against several types of cancers. However, administration of inappropriate doses of such TLR-modulating therapeutics or a wrong infusion administration is reported to induce detrimental outcomes. This review summarizes the current findings on the molecular and structural biology of TLRs and gives an overview of the potency and promises of TLR-directed therapeutic strategies against cancers by discussing the findings from established and pipeline discoveries.

## 1 Introduction

Cancer is the primary cause of human death with one out of six deaths worldwide (1). According to the World Health Organization (WHO), nearly 10 million deaths have been recorded in 2020 and the mortality graph is following an increasing trend (1). Various types of cancers have been diagnosed through the advancements in diagnostic methodologies. Regrettably, nearly every part of the human body is vulnerable to cancer development, and neoplastic growth in different organs is widely recognized as a major contributor to the lethal forms of cancer (1, 2). According to the reported statistics up to 2020, breast cancer, lung cancer, colon and rectum cancer, prostrate cancer, non-melanoma skin cancer, and gastric cancer respectively represent 2.26%, 2.21%, 1.93%, 1.41%, 1.20%, and 1.09% of the global disease burden of cancers (3, 4). In terms of mortality rate, lung carcinoma is reported as the most fatal type of cancer, followed by colorectal carcinoma, hepatocellular carcinoma, gastric carcinoma, and ductal carcinoma (3). Moreover, approximately four thousands children are usually diagnosed with cancers out of which cervical cancer is considered as one of the common forms of cancer (3).

With the effectiveness of immunotherapy in addressing various types of cancer in humans, the recent introduction of TLRs-based therapies is evidencing exciting results in terms of elongation of survival, and reduction of metastasis, as well as improving the overall prognosis of several diseases (5, 6). (Toll like receptors) TLRs are the pattern recognizing receptors (PRR) that play a fate-deciding role in various infectious and non-infectious diseases of humans including cancer (7–9). Due to this knowledge, TLRs are effective immunoglycoproteins that are activated through four different groups of ligands including damage/danger-associated molecular patterns (DAMPs), microbial/microbe-associated molecular patterns (MAMPs), pathogen-associated molecular patterns (PAMPs), and xenobiotic-associated molecular patterns (XAMPs) (10–13).

TLRs act as innate immune receptors and selectively bind to pathogenic ligands commonly known as PAMPs to elicit an innate immune response by activating the inflammatory signaling cascade (8, 9). Up to now, 13 TLRs (TLR 1-13) have been discovered in mammals (e.g., in mice (TLR1-13) and humans (TLR1-10)) which are grouped into two categories, i,e. extracellular or cell surface (TLR1, TLR2, TLR4, TLR5, TLR6 and TLR11) and intracellular (TLR3, TLR7, TLR8 and TLR9) TLRs (7, 11, 14, 15). Each TLR possesses a structure with three distinct domains viz. extracellular leucine-rich repeats (LRR) domain, a transmembrane domain, and an intracellular TIR domain (8, 9). The extracellular domain of a particular TLR binds to a specific pathogenic ligand through the coordinated association of a co-receptor namely MD2 (9, 16). Binding with the pathogenic ligand results in a conformational change in the intracellular TIR domain that facilitates the recruitment of various adaptor molecules, including myeloid differentiation factor 88 (MyD88), TIR-domain-containing adapter-inducing interferon-β (TRIF), TIR domain-containing adapter protein (TIRAP)/MyD88 adapter-like (MAL), and TRIF-related adaptor molecule (TRAM)) (7). Subsequently, TIRAP recruits MyD88 with TLR2 and TLR4 in order to stimulate the cascade of events in transcriptional activation of nuclear factor-κB (NF-κB) and/or mitogen-activated protein kinase (MAPKs) (7, 9, 17). On the other hand, the binding of TRIF with the TIR domain of TLR3/4 induces the proliferation of interferon regulatory factor-3 (IRF-3), NF-κB, and MAPKs (7, 17). The binding of bacterial LPS with TLR4-MD2 complex elicits a signaling cascade either through MyD88-dependent or -independent pathways (7, 8). TLRs, except for TLR3, mediate the downstream signaling through MyD88, a adaptor protein that ubiquitously expressed in all the immune cells and several cancer tissues (18–20). Previous findings have demonstrated that MyD88 and its related signaling pathways play crucial roles in the progression and development of cancer-associated cells. Therefore, identifying aberrant MyD88 expression is employed to predict prognosis of various human cancers (e.g., lymphoid, liver, hepatic, gastric and colorectal cancers (18, 21, 22). Herein, we present a comprehensive overview of the molecular and immunological aspects of TLR-directed chemo- and immunotherapy against different human cancers by reviewing the significant contributions made till date by the scientific communities across the globe.

## 2 TLRs in cancers

The causes of cancers are indeed multiple and multifactorial that include the effect of exogenous mediators as well as disruption of homeostasis in the human cell and tissue system (23). However, the gain-of-function of protooncogenes and loss-of-function of tumor suppressor genes are the most critical parameters behind the oncogenic transformation events occurring in the human body (23). Though, environmental pollution, altered lifestyle, and food habits, exposure to ionizing and non-ionizing radiations, consumption of carcinogenic contaminants through food and drinks, infection of oncogenic viruses e.g., hepatitis B virus (HBV), hepatitis C virus (HCV), human papillomavirus (HPV), and oncogenic bacteria e.g., *Helicobacter pylori* (*H. Pylori*)*, Fusobacterium nucleatum* (*F. nucleatum*)*, Clostridium* spp*, Escherichia coli* (*E. coli*) are the major causes of neoplastic transformations in humans (23–30). Moreover, human immunodeficiency virus (HIV) is known as an important biological predisposing factor for developing cervical cancer which may increase the risk of this cancer up to six-**folds** (31, 32). Nevertheless, several other agents as listed by the International Agency for Research on Cancer (IARC), and some genetic and physiological predisposing factors are also considered the risk factors for various cancers of humans (2, 33). So far, several prevention and intervention strategies have been implemented and are currently employed to reduce the harmful impact of cancer (5, 6, 34). Numerous prevention approaches have been recommended for avoiding or reducing the risk of cancer, and/or prolonging the survivability of cancer-affected individuals. These include quitting smoking and limiting alcohol intake, increasing physical **activities**, following a healthy diet, and maintaining a healthy lifestyle. On the other side, anticancer therapy is majorly comprised of the administration of **various** anti-tumor drugs/hormones, **TLR** agonists, and antagonists as immunomodulators and/or immunotherapeutics against various types of cancers (6, 35–37).

As discussed in the previous section, the TLRs belong to the PRR family recognizing the DAMPs, MAMPs, PAMPs, and XAMPs to elicit immune responses (11, 12). They are the key immune sensors for recognizing invading pathogens and are expressed over the sentinel of immune systems that includes macrophages and dendritic cells (7, 16, 38). TLRs play a vital role in the initiation and proliferation of malignant tumors, and in the prognosis of cancer. They promote the carcinogenesis process through the release of proinflammatory cytokines and anti-apoptotic factors, recruitment of immune cells, and proliferation of the cells across the tumor microenvironment (TME) to create a tumor-friendly milieu (39).

**Figure 1:**
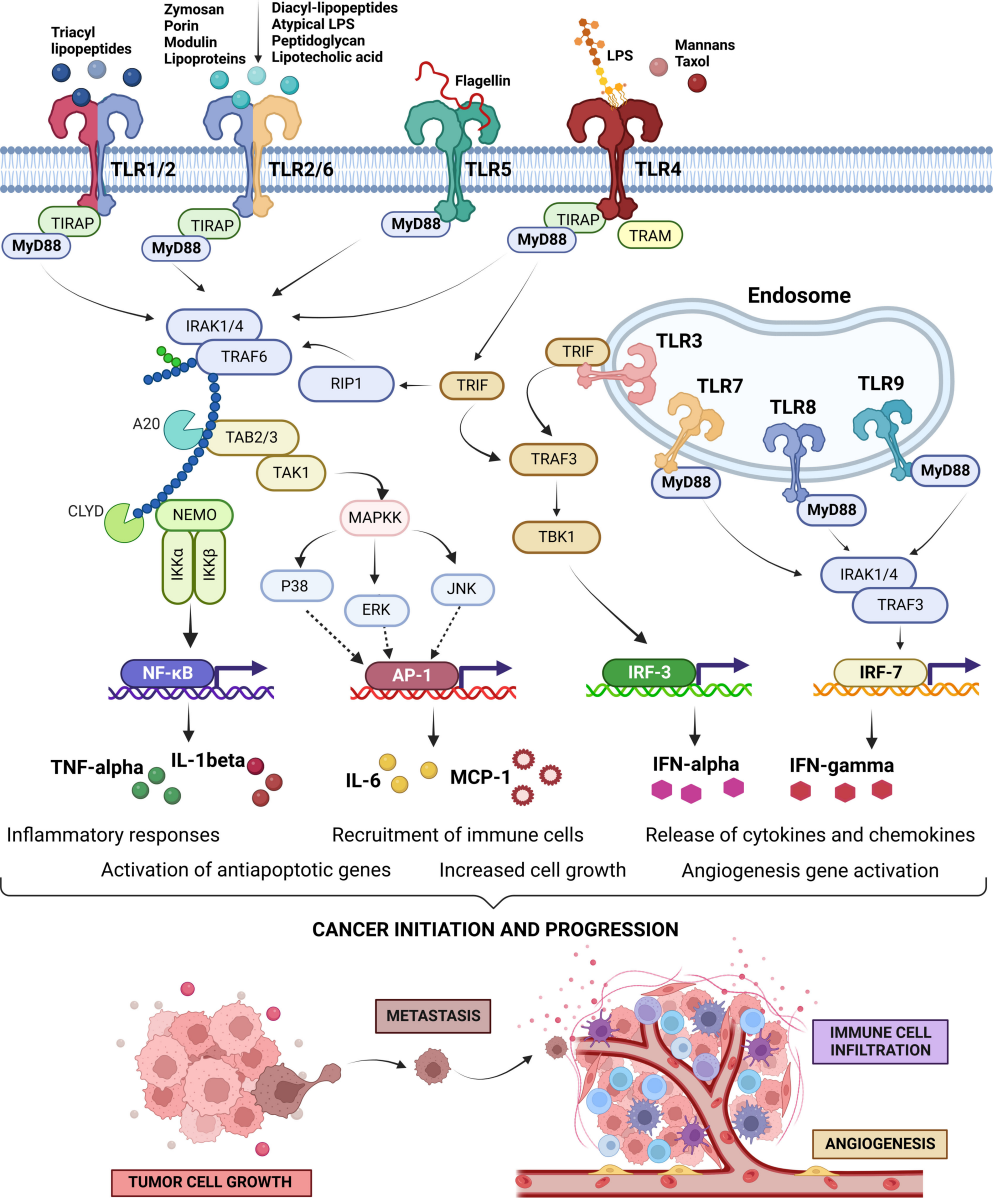
Toll-Like Receptors (TLRs) signaling pathways, intra-, and inter-signaling crosstalk circuits, and their regulatory loops. Cell surface expressed TLRs, including TLR1, 2, 4, 5, 6, 10, and 11, and intracellularly expressed TLRs on endosomal membranes, including TLR3, 7, 8, and 9, recognize their specific pathogen-associated molecular patterns (PAMPs) to activate their signaling pathways. Small molecules and/or microbial ligands, depicted on the top of the cell surface expressed TLRs, occupy the extracellular domain of the TLRs. Upon activation, they induce conformational changes in the intracellular domain to induce a signaling pathway by recruitment of the adaptor molecules (i,e., MyD88, TIRAP, TRIF, and TRAM) followed by downstream signals leading to the activation of sevral transcriptions factors (i.e., AP-1, NF-κB, IRF-3 or IRF-7). These key transcription factors drive the expression of several proinflammatory cytokines as well as antiapoptotic factors. Pro-inflammatory cytokines can promote cancer progression in three ways; firstly through facilitating immortalization by activating the proapoptotic factors, secondly recruiting the immune cells for creating a tumor-friendly microenvironment, and lastly by expanding the blood vessel to maximize the chances of metastasis (52–55). The image has been created with Biorender.com (56).

Moreover, TLRs are also associated with angiogenesis, metastasis and chemoresistance, and poor survivability. For example, the sensing of bacterial LPS mediates the activation of TLR signaling pathways resulting in hyperinflammation that promotes the pathogenesis of bacterial infection-induced carcinomas including gastric cancer, colorectal cancer, and lung cancers (40). The succession of events up to chronic inflammation is one of the hallmarks of the tumorigenesis process. The overactivation of the inflammatory cells leads to the secretion of growth and survival factors, overexpression of extracellular matrix remodeling enzymes, proangiogenic factors, and other reactive oxygen species (ROS) which in turn facilitate the mutagenesis, tumor growth, and invasion (5). Initially, TLRs were discovered as the component of the innate immune defense system but later on, TLRs were found to be ligated with antibodies to induce the expression of certain genes related to adaptive immune response (41–44). As already outlined, the TLR-mediated inflammatory pathways are routed through the MyD88-dependent and MyD88-independent pathways leading to the transcriptional activation of NF-kB signaling pathways (8, 9). Activated NF-kB (p65/p50 dimer) acts as the transcription factor to induce the secretion of the major proinflammatory cytokines like interleukin (IL)-1β, tumor necrosis factor-α (TNF-α), and IL-6 which promote carcinogenesis as well as metastasis (8, 9, 39). Apart from inducing inflammatory responses, TLRs also exert an antiapoptotic effect on the tumor cells that contribute to cancer progression (45–47). Apoptosis is an ordered and orchestrated biological process regulating cellular homeostasis by programmed cell death (48, 49). Immortalization of cells through acquiring resistance to the apoptotic process is another hallmark of cancer development (50). It is intriguing to note that, NF-κB is known to regulate the expression of anti-apoptotic genes and restricts the activation of pro-apoptotic pathways. TLRs upon binding to their respective ligands can directly activate NF-κB and/or the proinflammatory cytokines like IL-1β and TNF-α. The cytokines produced upon activation of the TLR signaling pathway also induce the activation of NF-κB, which subsequently triggers the expression of apoptotic factors. These factors have been found to promote the survival of tumor cells across different types of cancers (6, 51). The schematic representation of the cross-talks between the TLR signaling pathways in the course of initiation and progression of cancer cells is shown in **Figure 1**. The roles of different TLRs in promoting cancer development for different types of human cancers are discussed in the subsequent sub-sections.

### 2.1 Brain and neural cancer

Brain and neural cancer are referred to the tumors associated with the different parts of the central nervous system including both the brain and the spinal cord. The different types of cancers associated with the brain include astrocytomas, meningiomas, oligodendrogliomas, and mixed gliomas as the most common types, while ependymomas, mixed glial and neuronal tumors, and primitive neuroectodermal tumors as the less common ones (57). In this regard, experimental evidence on the increased cell surface expression of TLR1, TLR2, TLR4, TLR5, and TLR6 in astrocytoma samples compared to non-neoplastic brain tissues clearly revealed the involvement of these TLRs in the progression of astrocytoma (58). In fact, the activation of the TLR canonical pathway through TIRAP-MYD88 was found to induce the activation of NF-κB followed by the activation of JUN, and SRF which function as critical transcription factors to promote cellular proliferation (59). Earlier researchers have documented TLR2, 4, and 9 for their role in promoting tumors in glioma cells (60–62). In particular, the expression of TLR4 was detected at a higher level in U118, U87, A172, and LN229 glioma cell lines, and such high expression was linked with the regulation of cell growth and survival of the tumor cells (62). However, TLR4 expression was observed to be lower in glioblastoma (GB) tumors when compared to astrocytomas, and such downregulated level of TLR4 was reported both in chemoresistant GB and in macrophages co-cultured with GB cells (63). This behavior suggests the real strategy for GB-associated immune escape by the reduction of phagocytic functions of the macrophages that are normally induced by TLR4 (64).

Considering the intracellular TLRs, TLR9 expression was detected in the TME after radiotherapy and this finding suggests the beginning of the cancer recurrence process (65). In particular, TLR9 is known to play a key role in the formation of glioma stem cells (GSCs) (38). A study by Zang et al. (66, 67) demonstrated that adenovirus (ADV) infection could increase GSC formations by triggering the TLR9-MYD88 signaling in a STAT3-dependent way. Considering the metastasis stage, TLR2 triggers the NF-κB activation to signal the induction of the proinflammatory responses and overexpression of type 1 matrix-bound metalloproteinase (MT1-MMP) in microglia thereby activating tumor-released MMP2 that promotes metastasis (60, 61).

In children, medulloblastoma is known as one of the primitive neuroectodermal tumors with higher incidence and poor prognosis (68). It is widely heterogeneous, and its histopathological classification includes four subtypes such as classic, desmoplastic/nodular, medulloblastoma with extreme nodularity, and anaplastic (69). Interestingly, TLR7, TLR8, and TLR9 genes were found to possess a differential expression pattern in most of the common pediatric medulloblastoma histological subtypes (42). In contrast, a significant reduction in the expression of TLR7 and TLR8 was observed in the anaplastic subtype (42). The high expression of TLR7 was reported to ensure the best survival outcome after 60 months of follow-up than the low TLR7 expression (70). Considering the prominent and contrasting expression profiles, TLR7 has been proposed as a prognostic factor of survival in pediatric medulloblastoma patients.

### 2.2 Head and neck cancer

Head and neck cancer includes the cancer subtypes associated with the oral cavity, pharynx, larynx, nasal cavity, paranasal sinuses, and salivary glands. Oral and pharyngeal cancer are jointly the sixth most common type of cancers that occur through the prolonged use of tobacco through chewing and smoking, and alcohol consumption (71). In this direction, TLRs are known to play a vital role in the initiation and progression of oral squamous cell carcinoma and other cancers. TLR2 is expressed over the keratinocytes in oral squamous cell carcinoma and this receptor particularly regulates the growth and survivability of the tumor cells by promoting the immune-escape and inhibition of apoptosis (72). Considering the extent of expression, TLR3 is overexpressed in both head and neck carcinoma and squamous cell carcinoma (45). Mutations in the TLR3 genotypes are also found to be linked to the occurrence and development of carcinoma and are associated with the poor survival of the affected patients (73). In addition to TLR3, TLR4 has been reported to be a critical mediator of the tumorigenesis process in head and neck cancer (43). In fact, inflammatory cytokines, chemokines, and several other growth factors generated through the signaling cascade originating through the TLR4-MyD88-NF-κB axis regulate tumor growth as well as infiltration of the immune cells (71). In oral carcinoma, upregulation of TLR4 expression is found and such elevated level of TLR4 drives the transition of epithelial to mesenchymal cells to promote metastasis, cancer differentiation, and proliferation leading to poor survival and increased disease severity (74). Several studies suggested that the expression of TLR5, 7, and 9 is associated with poor differentiation and poor prognosis of oral cell carcinoma (71, 75, 76).

### 2.3 Esophageal cancer

Esophageal cancer is the eighth most common cancer globally with predominant cases of esophageal squamous cell carcinomas (ESCC) and esophageal adenocarcinoma (EAC). The normal human esophageal epithelial cells show a basal-level expression of TLR2, 3, 4, and 7 (77). However, a significant increase in the transcriptional expression of TLR3, TLR4, TLR7, and TLR9 is seen in ESCC (50). On the other side, elevated expression of TLR1, TLR2, TLR4, and TLR6 are usually observed in case of EAC (78, 79). *Ex vivo* experiments have shown that LPS stimulation of TLR4 in Barrett’s esophagus (BE) epithelial cell lines leads to an increase in cyclo-oxygenase 2 (COX-2) activity and an inflammatory response (65, 66, 80). Furthermore, activation of the TLR4-MyD88-TRAF6-NF-κB axis has been found to increase cell proliferation in EAC (65, 66). A high expression level of TLR5 was also found during the progression from metaplasia-dysplasia to EAC, while the expression of TLR9 was associated with advanced disease, poor differentiation, and metastasis (81). Further, high TLR3, TLR4, and TLR9 expression were also observed in stromal cells of ESCC and these were found to be associated with lymphatic metastasis, whereas the increase expression of TLR7 and TLR9 were noted to be linked to the advanced clinical stage of the disease (82, 83).

### 2.4 Lung cancer

Lung cancer is the most rapidly growing malignant tumor and currently contributes to the second largest cancer-related death worldwide. Non-small cell lung cancer (NSCLC) is the predominant subtype of lung cancer and is associated with 80% of cases (84). The physiological architecture of the transformed lung tissue itself indicates the involvement of inflammation in the oncogenesis process. Similar to other human cells, the airway epithelial cells of human lungs also possess various TLRs in their cell membrane and cytoplasm (14, 85, 86). These TLRs are known to play a role in amplifying inflammatory processes that contribute to tumorigenesis. The epigenetic regulation of TLR2 and TLR3 by DNA methylation has been reported to cause transcriptional activation leading to increased disease susceptibility and severity in lung cancer patients (84). On the other side, activation of TLR4 stimulated by the LPS has been seen to induce the activation of several vital cancer-critical signaling pathways that include the primary TLR4/NF-κB signaling pathways for inducing anti-apoptotic response and PI3K/Akt signaling pathways for uncontrolled cell division (37, 84). These signaling events are also implicated in the increased proliferation of lung carcinoma as well as poor disease survival (37, 84). The roles of different TLRs in the development, progression, and immunopathology of lung cancer have been extensively studied at the level of cytokines and chemokines. It is intriguing to note that the tumorigenesis and metastasis of different forms of lung carcinoma are principally mediated by the TLR2, TLR4, and TLR9-guided production of anti-inflammatory cytokines such as transforming growth factor-β (TGF-β), and IL-10 and growth factors such as vascular endothelial growth factor (VEGF) and fibroblast growth factor 2 (FGF2) (87). Moreover, the extracellular matrix remodeling and EGFR-mediated signaling axes induced by TLR2 and TLR4 create an optimal condition for the progression and metastasis of lung carcinoma (88). In particular, the ECM remodeling leads to the release of proteins such as fibrin and hyaluronan that may act as DAMPs for the PRRs including the TLRs to amplify the intensity of various inflammatory responses (89). Experiments conducted using human lung cancer *in-situ* and human lung cancer cell lines (e.g. A549) revealed that two intracellular TLRs namely TLR7 and TLR8 primarily signal the activation of the NF-κB signaling pathway, upregulation of the antiapoptotic protein Bcl-2 and the increase of tumor cell survival and chemoresistance processes (90). Many proteins, particularly NF-κB can regulate the non-canonical Wnt pathway by WNT5A ligand that functions as a “bridge” between the TLRs and Wnt signaling (91). Generally, WNT5A is an oncogenic protein involved in invasion and metastasis processes that increases its expression in human bronchial epithelial cells after exposure to cigarette smoke (92, 93). At the same time, tobacco smoke could induce the expression of TLRs in macrophages becoming both a very potent inducer of lung cancer and a trigger factor contributing to the unbalancing of Wnt/TLR (94, 95). TLRs have been discovered to be potential prognostic markers for lung cancer. Particularly in early-stage disease (i.e., stage 1), NSCLC and adenocarcinoma (ADC) patients display high mRNA expression of TLR1-10 and such overexpression is linked with improved overall survival of the patients (96). Moreover, a study using NSCLC patient serum demonstrated a significant correlation between the low level of soluble TLR4 with the poor survival of early-stage NSCLC (63). These aforementioned studies collectively support the proposition of TLRs as critical mediators and prognostic markers for NSCLC (97). In contrast, in advanced-stage NSCLC patients, an elevated level of TLR7 was reported to be strongly associated with a poor clinical outcome of NSCLC (64). Moreover, the outcomes of the mentioned study suggested that TLR7 could promote an immune suppressive microenvironment that facilitates the promotion of the immune evasion capacity of the tumor cells (98). Furthermore, elevated level of TLRs is also known to induce the expression of several pro-tumorigenic microRNAs (miRNAs) in the lung tissues. For example, a high level of TLR4 and TLR9 was found to be correlated with an increase abundance of miR-21 and miR-26a expression respectively (65). In fact, the upregulation of miR-21 and miR-26a was found to promote and increase the weight and size of the tumor mass in mice as well as the proliferation and migration of primary human lung cancer (99). An opposite behavior was observed for miR-15a/16 and TLR1 where overexpression of these miRNAs was seen to enhance the radiation sensitivity and overcome the radioresistance of the lung cells by regulating the TLR1/NF-κB signaling pathway (100). These results suggest the possibility of devising novel therapeutic strategies for lung cancers by either regulating the expression of the miRNAs by targeting TLRs or by modulating miRNA expression.

### 2.5 Breast cancer

Breast cancer is the most common type of cancer in America and is second in cancer-related deaths of women. It is a heterogeneous disease with multiple characters and clinical outcomes. Alike other cancers, mounting evidence suggests an important association between TLRs and the occurrence and development of breast cancer. TLRs play a predominant role in the TME of breast cancer and its pathogenesis. Studies conducted in malignant MDA-MB-231 cells suggest that the expression of TLR2 decreases up to 10-folds (67). However, breast cancer cells display activation of NF-κB and upregulation of expression of IL-6, TGF-β, VEGF, and matrix-metalloproteinase 9 (MMP9) (101). This evidence does suggest the involvement of other TLRs in the tumorigenesis process. In this context, TLR3 has been found to play an immunosuppressive role in the progression of cancer and survival, but its overexpression leads to increased metastasis (102). Moreover, dysregulation of TLR2 has been reported as a prognostic biomarker in breast cancer by implying a dual role in carcinogenesis and chemoresistance (103). Experimental stimulation of TLR2 in breast cancer stem cells (CSC) induces the activation of the MyD88/NF-κB and Akt pathways following the secretion of several cytokines (TGF-β and IL-6) and growth factors (epidermal growth factor (EGF)) that enable the cancer cells to survive and invade the nearby tissue or blood vessel (104). This behavior is in line with another observation that showed an increase in the expression of TLR2 in breast cancer lines endowed with high metastatic ability (101). The chemoresistance ability of TLR2 is expressed with a low relapse-time in anticancer chemotherapeutic-treated breast cancer patients and with a decrease in the sensitivity of several breast cancer cell lines to doxorubicin (103). In particular, the doxorubicin-induced immunogenic cell death releases TLR2-activating DAMPs, such as HMGB1, able to protects breast cancer cells from chemotherapy and promotes metastasis formation (105). Regarding TLR3, it plays an antithetical role in the progression of breast cancer and survival (106). The overexpression leads to an increase in metastasis and promotes both the CSC phenotype through the activation of Wnt/β-catenin and NF-κB signaling pathways and the mammosphere-like structure in breast cancer cells (107). On the contrary, upon stimulation with its ligand Poly(I:C), TLR3 induces a strong TRIF-dependent production of IFN-β, NF-κB activation, and finally the release of the pro-apoptotic cytokines and cytokine (IL-1β)-driven activation of the caspases (e.g. caspase-1) (102). Notably, TLR4 is highly overexpressed in breast cancer cells and promotes inflammatory response by stimulation from cellular ligands (e.g. HMG1) and results in increased cell proliferation, and lymph node metastasis, regulating the expression of integrin αvβ3 results in the adhesion and invasiveness of metastatic breast cancer cells (108, 109). In particular, it was observed that the expression levels of TLR4 and MyD88 are related to the metastatic and invasive potential of the breast cancer cell type. As we know TLR4/MyD88 signaling plays vigorous roles in several cancers. Wu et al. (93) showed that the expression levels of TLR4/MyD88 were positively correlated with the metastatic potential of breast cancer cells and tumors. These group further confirmed that the expression of the TLR4 is the key regulatory factor that determines invasiveness of cancerous cells from breast tissue (110). Additionally, the expression level of MyD88 alongside TLR4 was also found to be positively correlated with axillary lymph node metastasis and histological-grade breast cancer development (110).

### 2.6 Pancreatic and Hepatocellular cancers

Pancreatic cancer is the most lethal type of gastrointestinal tract cancer with the highest morbidity and mortality rate (111). The primary risk factors of pancreatic ductal adenocarcinoma (PDAC) include patient age, smoking status, obesity, diabetes, and status of chronic pancreatitis. These, subsequently lead to chronic inflammation which stimulates the TLR signaling pathways. TLR2 and TLR4 are usually found to be overexpressed in the pancreatic carcinomas tissues leading to poor disease prognosis and increased metastases (112). Apart from the cell surface TLRs, intracellular TLRs also play a fate-deciding role in the oncogenesis of pancreatic cancer. Viral infections cause stimulation of TLR7 and TLR8 through the viral ssRNA and/or other nucleotide ligands leading to immune cell activation and proliferation (78). Notably, inflammatory cytokines and other growth factors generated through TLR7 and TLR8 resulted in increased progression of metastatic cancer cells and downregulate the cell cycle regulators including cyclin D1, p16, PTEN, and the upregulation of p27, p53, p21, cyclin B1, PPARγ, and TGF-β (113, 114).

Hepatocellular carcinoma (HCC) represents the most frequent visceral neoplasm, occupying 70–90% of all primary liver cancer, and is characterized by heterogeneous malignancy, which happens via distinct pathway activation and molecular alterations (115, 116). Actually, the major treatment strategies against HCC are surgery, transplantation, and percutaneous ablations (117). The TME plays a critical role in the initiation, growth, and dissemination of HCC (118). An emerging evidence suggests a correlation between TLR activation and immune cell infiltration in HCC (119). Consequently, the dysregulation of the TLRs in HCC might contribute to tumor progression as observed by Liu et al. (120), where they identified several HCC-specific TLRs. In particular, a TLR-based gene signature (including MAP2K2, IRAK1, RAC1, TRAF3, MAP3K7, and SPP1) was identified in order to create an advantage in tumor prediction and to assist clinicians in selecting personalized therapy for HCC patients. To date, most studies have focused on the involvement of TLR2, 4, and 9 in the development of HCC. TLR4 and 9 have been reported as the critical factors in the progression of non-alcoholic fatty liver disease (NAFLD) and involve in mediating neutrophil dysfunction in cases of alcoholic hepatitis (121). Despite not receiving a thorough investigation, the TLR2 plays a critical role in liver disease progression, HCC development, and maintenance. Recently, a strong positive correlation was reported between cell proliferation index, the cytosolic expression, and nuclear translocation of TLR2 and apoptotic marker Caspase-3 expression particularly in HCC patients (121). Previously, it was shown how miR-143 downregulates TLR2 expression in hepatoma cells, leading to the inhibition of hepatoma cell proliferation (122). Additionally, TLR2 and TLR9 are involved in alcohol-induced liver injury by inducing CXCL1 and promoting infiltration of neutrophils (121). Investigations of the role of TLRs have uncovered various regulatory mechanisms that contribute to the increased production of pro-inflammatory and oncogenic molecules like cytokines (e.g., NANOG, Caspase-1, Ephrin-A1, NO, and BCL6). Thus, dysregulation of TLR4 has been associated with invasiveness and metastatic potential, as well as poor prognosis for individuals with HCC (123–125). Recent research demonstrated that high expression of TLR4 was associated with microvascular invasion in HCC (126). These observations indicate that TLR4 displays critical roles in HCC progression and the ability of miR-122 to modulate the innate immunity by blocking TLR4, which underlines the important role of this TLR in hepatocarcinogenesis (127). Moreover, TLR4 mRNA expression has been shown positively correlated with IL-6 and IL-10 mRNA expression and this correlation was stronger in obese HCC patients (128). Additionally, alongside TLR9, TLR7 has been identified as a pivotal regulator in tumor progression and is highly regulated in human HCC tissue (129). Their inhibition with oligonucleotide IRS-954 or chloroquine could potentially be used as a novel therapeutic approach for HCC development and/or progression. Finally, the TLR5 could represent an independent prognostic marker in HCC. Its localization, cytoplasmic or nuclear, is associated with high or poor 5-year overall disease-specific survival, respectively (130). These findings suggest a possible link between the TLR5 expression and the prognostic risk factors Ki67 and p53 in HCC progression (131).

### 2.7 Gastric cancer

Despite the declining incidence, gastric cancer (GC) is one of the most common malignancies worldwide. *Helicobacter pylori* (*H. pylori*) is by far the most important risk factor for GC development which is associated with early-onset cases (132–136). Although *H. pylori* infection triggers chronic inflammation by mediating immune regulators through pro-tumorigenic activities, its actual molecular pathogenesis in GC development is largely unknown. It is believed that *H. pylori*-associated carcinogenesis initiates gastric mucosal disturbance followed by chronic gastritis through a plethora of different signal transduction processes (137–139). In this scenario, diverse ligands of microbial and host cells have been identified to orchestrate various inflammatory responses leading to an interplay between *H. pylori*-induced chronic inflammation and the inflammatory milieu of the TME. Previous research has shown that the gastric mucosal immunity reacts to *H. pylori* infection by inducing the expression of TLRs, resulting in an inflammatory microenvironment generation (140, 141). Their involvement is essential for *H. pylori* recognition and subsequent innate and adaptive immunity against this bacterium. The human immune system crosstalk with *H. pylori* through several PRRs has been extensively reviewed by Cheok et al. (142).

Given their complexity roles in tumor immunity as either pro-tumor, anti-tumor, or dual effects, several TLRs (i.e., TLR2, 3, 4, 5, 7, and 9) have been reported to be dysregulated in human GC cells (142). Among them, increasing expression levels of TLR2, 4, 5, and 9 have been reported to be associated with cancer progression from normal gastric mucosa to pre-cancerous lesions, gastric dysplasia, and ultimately to gastric adenocarcinoma. Their overexpression suggests that TLRs may play a specific role in GC development (143).

Throughout the last decade, TLR2 and TLR4 have turned into an emerging candidate, that acts as critical innate immune sensor to trigger the inflammatory response to many ligands of microbial and their targeted host. Notably, several studies have shown that TLR1, TLR2, TLR4, and TLR10 gene polymorphisms are associated with increased GC risk, and its expression is significantly raised in *H. pylori*-positive gastritis patients as well as GC patients (144–147). Some of these TLRs, TLR1, 2, 4, 5, and 6, recognize different membrane components like lipids, lipoproteins, and proteins, binding their ligands on the cell surface, while others, including TLR3, 7, 8, and 9, play a major role in the recognition of microbial nucleic acids, which are identified in the extracellular vesicles. It has been shown that TLR2, 4, and 5 subtypes are critically involved in immune responses to bacterial infections, being abundantly expressed in immune cells. In general, TLR2 recognizes PAMPs mainly from Gram-positive bacteria, TLR4 is the receptor for Gram-negative bacterial lipopolysaccharide (LPS) and TLR5 recognizes bacterial flagellin (148). Moreover, TLR3 (regarded as a potential therapeutic target for multiple cancers), TLR5 (an effective target for antitumor immunotherapy), and TLR9 (involved in both anti-tumor and pro-tumor responses) are overexpressed in GC that lead to increased cell proliferation, dysplasia, metaplasia, lymph node metastasis, and poor survival (149, 150). Lastly, TLR7 and TLR8 were also shown to recognize purified *H. pylori* RNA that mediates the IL-6/IL-12 response, whereas TLR9 recognizes unmethylated CpG DNAs.

Additionally, there is growing evidence that host-derived RNA species (e.g., miRNAs) could interact with TLRs (151, 152). Being involved in regulation at a post-transcriptional level as either oncogenes or tumor suppressors, miRNAs can either inhibit the translation or facilitate the cleavage of their targeted mRNA. Moreover, miRNAs gained great interest for their potential use as biomarkers in several human diseases (153–155). It has been reported that induction of miRNAs by TLR ligands could affect TLR pathway activation and initiate the signaling cascade of immune response (e.g., through stimulating NF-κB signal downstream to TLRs) (156, 157). Given the importance of LPS–TLR interactions in *H. pylori* pathogenesis, miRNAs serve multiple regulatory functions in infection pathogenesis. Accordingly, differential expression of several miRNAs (e.g., miR-9, miR-105, miR-146a, miR-132, and miR-212) have been reported to negatively regulate TLR2-induced cytokine production, thereby fine-tuning the immune system (156–158). Therefore, the interactions between miRNAs and TLRs as a common language of “cell-to-cell communication” are associated with the prognosis for and progression of multiple human diseases, including cancer.

As already outlined, *H. pylori*-associated pathogenesis is linked to the severity of the host inflammatory response. Although the actual role of TLRs in LPS recognition is still contradictory, the binding of *H. pylori* LPS with TLR2 and TLR4 initiates the proinflammatory responses to develop the early stages of GC. After bacterial recognition, both TLR2 and TLR4 are activated in cooperation with the adapter molecule MyD88, triggering the MAPK signaling pathway. Thereafter, these signaling cascades activate the transcription factor NF-κB and drive the secretion of distinct cytokines i.e., proinflammatory cytokines including IL-1β, IL-2, IL-6, IL-8, and IL-12 (140, 159). Smith et al. (160) reported that as a classic TLR2 ligand, *H. pylori* LPS activates NF-κB and transcription from the IL-8 promoter and induces expression of a discrete pattern of chemokines such as CXCL1, CXCL2, CXCL3, and CCL20 through pathways involving MyD88, MAL, IRAK1, IRAK4, TRAF6, IKKβ, and IĸBα (160).

TLR5 is well-known for its role in recognizing bacterial flagellin, the bacterial structural protein that is expressed on the surface of epithelial cells as well as some innate immune cells. This propriety enables *H. pylori* to move between the mucus layer of the stomach and the surface of epithelial cells, therefore playing a key role in initial colonization (161). Previous research showed that live *H. pylori* or its purified flagellin activates the NF-κB pathway through binding to TLR5. Significant upregulation of TLR5 is also detected in THP-1 cells following *H. pylori* infection, causing secretion of IL-8 and TNF-α, which initiate inflammation (142).

The cag pathogenicity island (cagPAI) of *H. pylori* encodes a type IV secretion system (T4SS) which is associated with the gastric disease; being Cag3, CagM, CagT, CagX, and CagY proteins as the T4SS core complex of this bacterium (162). The T4SS-pilus protein CagL of *H. pylori* interacts directly with TLR5 (163). Enrolled in bacteria–host cell interface, T4SS pilus, a needle-like surface appendage is induced upon host contact resulting in better bacterial proliferation and persistent colonization of the body (164). Recent studies have reported that components of T4SS, such as CagL and CagY, in pathogenic *H. pylori* strains can serve as TLR5 agonists in driving the innate immune activation and recruitment of T helper 1 (TH1) cells (15, 150). CagY, a pilus-associated protein, has been identified as a strong flagellin-independent agonist of TLR5 leading to fundamental innate immune responses mediated by this pathogen (162). However, due to the low intrinsic activity of *H. pylori* flagellin, demands regarding which bacterial factors activate TLR5 are mounting.

In summary, GC is associated with heterogeneous pathophysiological criteria concerning anatomical location and histological subtypes. Among several associated factors (e.g., gastro-esophageal reflux, atrophic gastritis, male gender, smoking, and diet), its interaction with *H. pylori* mediates the production of inflammatory cytokines and chemokines. The binding of LPS of *H. pylori* with TLRs (TLR2, 4, and 5) initiates the proinflammatory responses that give rise to GC. Although their actual role in the evolution of gastritis remains unclear, TLRs have been associated with several cellular processes such as increased cell proliferation, dysplasia, metaplasia, lymph node metastasis, and poor survival in GC (149, 150). Overall, the existence of diverse bacterial ligands recognized by the TLRs, their complex role in innate and adaptive immunity, microbial interactions with other epithelial receptors of the gastrointestinal tract, host genetics, and environmental variables, require more investigations to better understand the causal role for specific TLRs in GC.

### 2.8 Colorectal cancer

Colorectal cancer (CRC) is the third most common cancer worldwide and is second in causing cancer-associated deaths (165). Chronic inflammation that maintains a favorable TME and systemic immune response are the key factors in the neoplastic transformation of colon tissue (111, 166). The intestinal epithelium expresses several TLRs that play a dual role in pro-tumor and/or anti-tumor activities in the case of CRC. Being enrolled in microbial-induced proteins, several TLRs are shown to be expressed in most epithelial cell lineages; among them, TLR1, 2, and 4 bind to MyD88 followed by activation of NF-κB by binding to interleukin-1 receptor-associated kinases (IRAK1, 2, and 4 (167).

So far, the dysregulated expression of several TLRs has been reported in CRC patients either in cancerous or noncancerous tissues (168). The upregulation of TLR2 and TLR4 genes has been a subject of considerable interest in CRC research. Their expression levels vary depending on the stage of the disease, with higher expression levels being associated with more advanced stages of CRC (169–171). TLR2 shows differential expression across the different clinicopathological conditions of colon carcinoma. It increases tumor formation by elevating the level of IL-6, IL-17A, and STAT3 (51). However, in colitis-induced cancer, TLR2 shows antitumor activities (172, 173). In this context, TLR4 plays an active role in the colon by maintaining immune homeostasis. A study by Wang et al. (174) suggested that the overexpression of TLR4 and MyD88 in the gut tissue resulted in gut inflammation and infiltration of immune cells that contributed to the tumorigenesis and progression of CRC with a higher degree of metastasis and poor survival. In stromal fibroblast, the expression of TLR4 is associated with a poor prognosis of CRC (174).

As already outlined, TLRs, particularly TLR2 and 4, are highly expressed in human rectal adenocarcinoma cells and serve as receptors for PAMPs. These two TLRs are the best-characterized PRRs which identify either invading pathogens outside the cell and/or intracellular pathogens engulfed in the endosomes or lysosomes (8, 152). Without going into the details of their pathogenesis, it has been identified that colonic adenomas and adenocarcinomas are colonized with diverse microbiota that play fundamental roles in intestinal homeostasis and disease progression. Therefore, it is believed that alterations in the gut microbiota followed by their excreted metabolites are closely related to CRC progression (153, 167, 175, 176). Indeed, this alteration may affect the expression pattern of TLRs on the epithelial cell surface leading to increased intestinal permeability and distinct features of “microbial dysbiosis”. Thus, any imbalance in the composition of the gut microbiota, as well as epithelial cells, may disturb immune homeostasis such as TLR signaling pathways, leading to uncontrolled inflammation and disease progression (40, 177–180). For example, *Enterotoxigenic Bacteroides fragilis*, *Fusobacteria* spp., and pks+ *E. coli* have been identified in association with the development of adenoma and/or CRC (26, 27, 181, 182). However, regardless of accumulating evidence of microbial composition in CRC pathogenesis, a better understanding of the crosstalk between tumor-associated bacteria and TLRs is required (40, 177). Overall, the mechanistic insights of the oncogenic transformation of normal colon tissue to CRC via activation of the TLRs still need some attention from the scientific communities.

### 2.9 Ovarian cancer

Ovarian cancer is a highly devastating and life-taking gynecological cancer characterized by the neoplastic transformation of ovarian epithelial, stromal, or germline cells (183). The ovarian cancer microenvironment is highly immunosuppressive. It consists of elevated levels of IL-10, IL-4, and TGF-β, thereby suppressing the macrophages and dendritic cells and increasing VEGF (184). Several studies suggested the overexpression of TLR2, 3, 4, 5, and 9 throughout the carcinogenic ovarian epithelial cells which induce the metastasis process (185–188). The TLRs are expressed over the normal ovarian epithelial cells and induce inflammatory responses thereby eliciting the neoplastic transformation of the ovarian tissues. TLR3 mediated immune responses by triggering tumor cell growth and survivability and are associated with cancer progression (184, 188). Instead, TLR4 is stimulated through the binding of LPS over the tumor cells to increase the production of inflammatory cytokines that lead to inhibiting the ability of CTLs for cancer cell recognition and death (185, 189). TLR4 overexpression is associated with its immunosuppressive role in disease progression and increased cancer cell survival (189). Studies showed that the increased expression of TLR4 in ovarian cancer results in resistance to several chemotherapeutic drugs, including Paclitaxel (187). Moreover, the high expression of TLR9 in ovarian cancers leads to increased disease severity, poor survival, increased tumor grade, and metastasis (34, 186). The role of different TLRs across different cancers is summarized in **Table 1**.

**Table 1:** Involvement of TLRs in inducing susceptibility and resistance to different human cancers.

Cancer(s)	Mode of involvement of TLRs	Associated TLRs	Pathological consequences and contributing factors	References
Brain and Neural cancer	Activation of NF-κB signaling and modulation of p38/MAPK pathway causing cell growth and viability; induce overexpression of membrane type 1 matrix-bound metalloproteinase (MT1-MMP) in microglia activating tumor-released MMP2 leading to metastasis	TLR2	Increased expression in glioma biopsies; decrease patient survival; promote tumor growth	(60–62)
	Promotion of tumor growth mediated via the inflammatory signaling pathways	TLR4	Higher protein expression in U118, U87, A172, and LN229 glioma cell lines, regulation of cell survival, immune infiltration, and tumor progression	(62)
	Trigger TLR9-MYD88 signaling in a STAT3-dependent way	TLR9	Glioma stem cell (GSC) formation	(67)
Head and neck cancer	Expressed over the keratinocytes in oral squamous cell carcinoma	TLR2	Overexpressed significantly across the primary tumors, regulate apoptosis of tumor cells	(72)
	The mutated genotype of TLR3 is associated with the development of carcinoma	TLR3	Overexpressed in head neck and oral squamous cell carcinoma; poor survival	(73)
	Induction of signaling cascade mediated via MyD88 inducing secretion of inflammatory cytokine, chemokines, and epithelial to mesenchyme transition	TLR4	Higher expression, tumor differentiation and proliferation, poor survival, and disease severity	(71, 74)
	Promotion of tumor growth mediated via the induction of inflammatory signaling pathways	TLR5, 7, 9	TLR5 expression is associated with a lower grade of tongue cancer; TLR7 is upregulated in oral carcinoma showing poor differentiation and prognosis; TLR9 shows higher expression in primary oral cell carcinoma but lower across squamous cell carcinoma	(71, 75, 76)
Esophageal cancer	Expressed in esophageal squamous cell carcinoma mediated through the activation of NF-κB	TLR3, 4, 7, and 9	overexpressed in esophageal squamous cell carcinoma; TLR3, 4, and 9 associated with lymph node metastasis; TLR7 and 9 expressions related to poor histological grade; TLR4 stimulation by LPS increases migration and adhesive properties	(79)
	Stimulation of TLR4 with LPS resulted in NF-κB activation and increased IL-8 secretion	TLR1, 2, 4 and 6	Overexpressed in EAC; TLR9 expression is associated with metastasis, poor grade of differentiation, and prognosis	(78)
Lung cancer	Epigenetic regulation and transcriptional activation. Production of anti-inflammatory cytokines	TLR2, 3	Promotes tumor growth and proliferation	(84)
	Activation by LPS stimulation leads to activation of the PI3K/AKT signaling pathway. Production of anti-inflammatory cytokines. Extracellular matrix remodeling and EGFR-mediated signaling	TLR4	increase in the proliferation of human lung adenocarcinoma cell line A549; upregulating anti-apoptotic protein Bcl-2 and downregulating pro-apoptotic protein; increased metastasis	(37)
	Activation of the NF-κB signaling pathway. Upregulation of anti-apoptotic protein Bcl-2	TLR7, 8	Increase in tumor cell survival and chemoresistance	(90)
Breast cancer	Promote tumor growth mediated via the inflammatory signaling pathways. Activation of the MyD88/NF-κB and AKT pathways. Release of TLR2-activating DAMPs, such as HMGB1	TLR2	TLR2 shows ten-fold lower expression in malignant MDA-MB-231 cells; TLR2 promotes the survival, invasion of these cancer cells, and induction of chemoresistance	(101–104)
	Induction of strong TRIF-dependent production of IFN-β, together with NF-κB activation. Activation of Wnt/β-catenin and NF-κB signaling pathways	TLR3	Release of pro-apoptotic cytokines and activation of caspases. For example, activation of caspase-1 from procaspase-1 is induced by IL-1β. Increase of metastasis and promotion of the CSC phenotype and mammosphere-like structure	(102, 107)
	LPS triggered increased expression of TLR4 downstream MyD88 signaling cascade to felicitate inflammatory responses	TLR4	Overexpressed, increased cell proliferation, lymph node metastasis; regulate expression of integrin αvβ3-mediated adhesion and invasiveness of metastatic breast cancer cells	(108)
	Induction of inflammatory responses through binding of TLR agonist(s)	TLR5, 9	Overexpressed breast cancerous cells promote cancer progression and poor survival	(109)
Pancreatic cancer	TLR2 binds with HMGB1 and activate PI3K/Akt as well as Wnt/β-catenin pathways for generating tumor-promoting milieu	TLR2	Overexpression; poor progression of disease; increased metastasis	(112)
	Activation of NF-κB and modulation of gene expression such as MMP2,9 in response to stimulation by LPS, leading to the expansion of the tumor cells	TLR4	Upregulation of TLR4 resulted in cancer proliferation, increased angiogenesis, metastasis, and disease progression and severity	(114)
	Stimulation by viral ssRNA and/or ligand leads to immune activation and proliferation	TLR7, 8	Increased in the progression from PanINs to metastatic cancer; downregulation of cell cycle regulators including cyclin D1, p16, PTEN and the upregulation of p27, p53, p21, cyclin B1, PPARγ, and TGF-β; resistant to chemotherapy	(113, 114)
Hepatocellular cancer	Nuclear translocation and activation of VEGF and Caspase-3 genes	TLR2	Upregulation of TLR2 increased cell proliferation and expression of vascularization markers	(121)
	Induction of stem-like features via activation of the TLR4/Nanog pathway	TLR4	Upregulation of TLR4 increased microvascular invasion	(126)
	Activation of NF-κB pathway as well as p-Akt expression	TLR7 and TLR9	Upregulation od TLR7 and TLR9 increased HCC cell proliferation	(129)
Gastric cancer	Interact with *Helicobacter pylori* and mediate the production of proinflammatory cytokines and chemokines	TLR3, 4, 5, 9	Interact with *H. pylori* to induce gastric carcinoma, overexpressed; increase dysplasia and metaplasia; lymph node metastasis, poor prognosis, and poor survival	(149, 150)
Colorectal carcinoma	Formation of heterodimers with TLR1 and/or TLR6 to initiate the signaling cascade for activating the transcription factors like NF-κB and AP-1	TLR2	Increased tumor formation and increased levels of IL-6, IL-17A, and STAT3; show antitumor activities	(93, 94)
	TLR4 is activated by bacterial LPS and lipoteichoic Acid (LTA). Upon recognition by LPS Binding Protein (LBP), it transfers to the differentiation-14 (CD14) receptor or MD-2, which are the accessory proteins involved in the ligand recognition, dimerization, and endocytosis of TLR4	TLR4	Activation of NF-κB through the MYD88 pathway, leading to transcription of pro-inflammatory cytokines as well as induction of Nox-derived ROS, resulting in tumor cell metastasis	(190, 191)
	TLR9 is located in the cytoplasm and intracellular endosomes recognize unmethylated CpG motifs in bacterial DNA	TLR9	Involved in colitis−associated colorectal carcinogenesis by regulating NF−κB expression levels	(192, 193)
Ovarian cancer	Binds to dsRNA and analogs to initiate a signaling cascade by activating NF-κB leading to the upregulation of IFN-α and IFN-β, CTL, and NK cells	TLR3	Overexpression and tumor progression promote cancer cell growth and survival; elevated production of cytokines (IL-6) and chemokines	(184, 188)
	Over the tumor cells, the LPS stimulates the TLR4 for the increased production of IL-6, inhibiting CTLs for cancer cell detection and death	TLR4	Overexpression in cancerous epithelial cells; immunosuppression; increased cancer cell survivability and tumor progression; development of chemoresistance to Paclitaxel	(185, 187, 189)
	Hypomethylated tumor DNA released from the tumor cells binds to TLR9 to trigger the signaling cascade promoting tumorigenesis	TLR9	Increased expression leads to disease severity, poor survival, increased tumor grade, and metastasis	(34, 186)
Hematologic Cancers	SNPs rs3804100 (S450S) and rs4696480 (16933T>A)	TLR2	Associated with marginal zone lymphoma (MZL), increases the risk of follicular lymphoma (FL), and decreases the risk of chronic lymphocytic leukemia (CLL). Its expression is also associated with poor prognosis in CLL patients	(194–196)
	SNP rs4986790 (A299G)	TLR4	linked with an elevated risk for MALT lymphoma. It could trigger a cascade resulting in Mantle cell lymphoma (MCL) growth and evasion from the immune system	(195, 197)
	SNPs rs5743836 (1237T>C) and rs352140 (2848 G/A)	TLR9	Associated with an elevated risk for NHL and linked with lack of cell death in the Mutu-I and BJAB Burkitt lymphoma (BL) cell lines. It could be a potential biological marker for the response to BL treatment	(198, 199)

Intriguingly, TLR gene polymorphisms, which alter the activities of the TLRs, result in the alteration of manifestations of many infectious and inflammatory diseases, including cancer (8, 9). For instance, the occurrence of GC has been associated with TLR4 polymorphisms viz., rs4986790 and rs4986791 (200). Similarly, TLR3 polymorphism rs5743312 has been found to increase the occurrence of oral cancer (201). A recent study by Hu et al. (202) revealed a relationship between TLR4 polymorphism and pan-cancer through an integrated omics approach describing that TLR4 gene expression is remarkably correlated with the expression of DNA methyltransferase 2 (DNMT2) and DNMT3B in the course of skin cutaneous melanoma and stomach adenocarcinoma.

### 2.10 Hematologic cancers

Hematologic cancers include several clonal malignancies (e.g., lymphoma, leukemia, and multiple myeloma) that arise from cells of the immune system at different stages of their differentiation process, starting from blasts to memory cells. Lymphomas are proliferative diseases of lymph nodes or extra-nodal lymphatic tissue, arising from B, T, or NK cells at distinct maturation and differentiation stages (203, 204). Lymphomas are divided into non-Hodgkin’s lymphoma (NHL), which contains the most prevalent forms of lymphomas, and Hodgkin’s lymphoma (HL), which is categorized into a classical (cHL) (represents approximately 95% of patients) and a nodular lymphocyte predominant form (NLPHL) (for only 5% of cases) (205, 206).

Accumulating evidence reported the aberrant expression of TLRs on the transformed cells of the immune system to contribute to the development of hematopoietic and related malignancies (207, 208). TLR gene variants have been reported as having potential functional relevance associated with lymphoma etiology and prognosis. By meta-analysis study of TLR gene polymorphisms in NHL, it was reported that variations in the TLR10, TLR1, and TLR6 regions are associated with NHL risk. In particular, two SNPs within the region, rs10008492, and rs4833103, were significantly associated with NHL and correlated to TLRs expression (194). Furthermore, the TLR2-rs4696480 variant has been shown to increase the risk of follicular lymphoma (FL), whereas it decreases the risk of chronic lymphocytic leukemia (CLL) (195). TLR4 Asp299Gly SNP has been related to lymphoma by elevating the risk for MALT lymphoma; an increased risk of HL and T-NHL. Moreover, among several TLRs expressed by Mantle cell lymphoma (MCL) cells, TLR4 is reported among the highest-expressed molecules. LPS upregulates the secretion of inflammatory cytokines leading to increased proliferation of MCL cells. Therefore, TLR4 signaling could trigger a signaling cascade resulting in MCL growth and evasion from the immune system (197). Other studies have also shown that TLR9-rs5743836 SNP is also associated with an elevated risk for NHL (198). This SNP has been recently reported to be linked with the Mutu-I and BJAB Burkitt lymphoma (BL), resulting in enhanced NF-κB activation upon TLR9 triggering (199). The association between TLR9-rs352140 SNP and cell death responses of BL cells highlights its potency as a biological marker and an anti-cancer agent (synthetic TLR9 agonists). Acute lymphoblastic leukemia (ALL) is another hematologic malignant disorder of lymphoid progenitor cells, and it is due to various significant genetic lesions in B/T-precursor-stage lymphoid cells, including mutations that induce aberrant cell proliferation and lymphoid differentiation arrest (209–211). As the most common pediatric malignancy, the B-cell precursor acute lymphoblastic leukemia (BCPALL) expresses detectable alterations in costimulatory molecule expression of TLR2, TLR7, and TLR9, being TLR2 ligands PAM3CSK4 and PGN, the most powerful effect on anti-ALL immune responses (212). In another study, the SNPs analysis on patients with newly diagnosed B-cell acute lymphoblastic leukemia (B-ALL) revealed the role of three specific TLR2 and TLR4 genotypes (TLR-2 Arg753Gln, TLR-4 Thr399Ile, and TLR-4 Asp299Gly) that could predict good B-ALL patients outcome (213). Multiple myeloma (MM) is a fatal B-cell malignancy determined by an accumulation of neoplastic plasma cells in the bone marrow (214). MM accounts for 10% of all hematologic malignancies and the incidence rate accounts for 0.8% of all cancers and its death rate indicates 1.0% of all cancer deaths per year worldwide (215). In addition to above mentioned myeloid malignancy, a broad spectrum of TLRs expression has been reported in MM patients. Among the most frequently expressed TLRs, heterogeneous expressions of TLR3, 4, 7, and 9 on primary myeloma cells have been reported (216). The canonical NF-κB pathway followed by the expression of IL-6 is among the most important growth and survival factors in MM cells (217). The expression level of TLRs mediates the autocrine loop secretion of IL-6 which has a pivotal role in the survival, growth, and drug resistance of MM cells (216). On the other hand, dysregulated TLRs could induce immune escape of MM cells through inhibition of NK cells (218) as well as induction of NF-κB-dependent proliferation of myeloma cells resulted in MM cell growth and proliferation (216).

Finally, B-cell chronic lymphocytic leukemia (B-CLL) is one of the most entirely studied forms of hematologic malignancies characterized by a progressive accumulation of monoclonal CD5^+^/CD19^+^, CD23^+^, CD21^+^, CD40^+^ B cells with prolonged cell survival and low proliferative index in the peripheral blood, bone marrow, and lymphoid organs (219). The cellular origin of CLL remains unclear, but numerous experimental data suggest that CLL results from a multiplication of B lymphocytes selected during clonal expansion through multiple exposures to antigens (220). Data regarding the TLR expression spectrum in B-CLL is associated with TLR2, 4, and 10. A lower percentage of CD19^+^/CD5^+^TLR2^+^ cells in patients with CLL compared to the control group was found and mean fluorescence intensity (MFI) data indicated that low TLR2 expression is associated with poor prognosis in CLL patients, suggesting that TLR2 could become a potential biological marker for the clinical outcome in patients with CLL (196). Skorka et al. (221) observed a significantly lower expression of splicing variants of TLR4 (TLR4 (1) and TLR4 (4)) in the peripheral blood mononuclear cells (PBMC) in CLL compared to healthy volunteers (HVs). They identified that the splicing variants of TLR4 (3) could impact clinical outcomes in CLL. Moreover, this group suggested a potential prognostic role of high expression of TLR9 mRNA in the bone marrow in CLL due to shorter time to first treatment (TTFT) in groups of CLL patients with high TLR9 expression in comparison to low TLR9 expression in bone marrow mononuclear cells (BBMC) (221). The TLR9 high expression was confirmed in autologous plasma from patients with CLL that contains a disproportionately high level of unmethylated mtDNA, able to trigger TLR9 signaling (222).

## 3 Tumor microenvironment and tumor immune microenvironment

TME is a feature obtained from an intimate communication between stromal- (e.g., different types of immune cells, endothelial cells, and cancer-associated fibroblasts (CAF)) and tumor cells. Indeed, TME is the outcome of an effective and dynamic network composed of chemokines, cytokines, soluble factors, adhesins, and growth factor molecules. The natural characteristics of the TME network may lead to tumor development via cell migration within the tumor mass, extracellular matrix remodeling, and the growth of aberrant lymphatic and vascular networks. As results show, the existence of chemotherapy-resistant cancer cells within the TME results in the occurrence of mutations which makes it an aggressive tumor (223–225). In brief, the active communications between immune biomolecules and tumor cells within TME support cancer development and progression (226, 227).

Activation of TLRs may lead to the induction of a cascade of biological activities such as TLRs signaling pathways and chronic inflammation-induced tumorigenesis. As previous recorded reports show, chronic inflammation is the pivotal stimulator of TME which results in the induction of tumor cell proliferation, survival, and suppression of anti-tumor immunity. Hence, the activation of uncontrolled TLRs signaling pathways is the unfavor edge of the TLRs double-edged sword (227–230). Indeed, a wide range of diseases e.g., autoimmune diseases, infectious diseases, cancers, etc. appear as a consequence of any dysregulations in association with TLRs signaling pathways. Therefore, blocking the TLRs involvement in inflammatory diseases and recruitment of TLRs signaling pathways in opposition to cancers are effective options that can be achieved through manipulation of TLR signaling pathways. Due to this knowledge, TLR agonists are recruited as vaccine adjuvants and antimicrobial agents; while the TLR antagonists are exploited as immunosuppressive drugs (13, 231).

TLRs are important biomolecules that can be expressed in different types of cells including immune cells, tumor cells, and tumor tissues. In this regard, the expression of TLRs and the rate of their expression within the human host body depending on the progression and condition of different tumor cells and cancers vary, entirely. TLRs have both pro-tumor (such as invasion, proliferation, migration, cancerous stem cells maintenance) and anti-tumor (induction of both innate and adaptive immune responses) effects in TME which shows the TLRs dual role in opposition to each other (62, 232, 233). Both necrotic cancerous cells and damaged epithelial cells (with the normal condition) produce a considerable amount of DAMPs (e.g., adenosine triphosphate (ATP) molecules, heat shock proteins (HSPs such as HSPs 60 and 70), nucleic acids, uric acid, HMGB1, Ca^2+^ regulatory S100 family protein) within the TME. DAMPs are known as important inducers of TLRs which activate the chronic inflammatory signaling pathways within the TME, evoke regulatory T (T reg) cells, myeloid-derived suppressor cells (MDSC), and M2 MΦs as immunosuppressive response. In parallel with the activation of chronic inflammation within TME, the process of progression and development of the tumor is going on via immune cells and in particular with the participation of the TLRs (200, 234–236).

The chronic inflammation feature in TME makes TLRs an effective inducer of inflammatory responses through the NF-κB signaling pathway that promotes cancer cell stemness. In the following, the appearance of further cancer stem cells promotes the induction of NF-κB. This process results in the development and progression of tumors. In addition, the triggered TLRs within tumor cells activate a cascade of processes such as enhancement of tumor cells proliferation, anti-apoptosis feature, enhancement of tumor cells invasion, metalloproteinases- and integrins-mediated metastasis, activation of biosynthesis pathways of pro-inflammatory factors and immunosuppressive biomolecules, promotion of cytotoxic lymphocyte-resistant tumor cells and increase in immune evasion. As previous studies show, the interaction of molecules involved in cellular energy metabolism in tumor cells and tumor-infiltrating immune cells within TME are modulated by TLRs. Moreover, regulation of TME occurs via the ubiquitination feature within TLRs signaling pathways (227, 237–239).

On the other hand, TIME is known as the properties of inflammatory responses and the composition of immune cells within a tumor. TIME is classified into two categories infiltrative exclusion (I-E) TIME (recognized as cold tumor because of inactivation of adaptive immune responses) and infiltrative inflammatory (I-I) (recognized as hot tumor because of high invasion of PD-1 producing cytotoxic T-cells, and PD-L1 expressing leukocytes and tumor cells) (227, 231). As mentioned about TEM, the released DAMPs via necrotic cells may induce TLRs which results in immune cell activation within the TIME. The DAMPs including HMGB1, HSPs, S100 proteins, etc. induce inflammatory responses through the NF-κB signaling pathway. The aforementioned DAMPs comprising HMGB1, HSPs, and S100 proteins activate inflammatory responses through the NF-κB signaling pathway through triggering the TLRs. This feature results in the activation of immune suppressive cells into TIME. Hence, DAMPs are able to support and induction of immunosuppressive TIME. Our depth understanding of these mechanisms and biological activities represents a bright promise to obtain considerable strategies and solutions for effective and powerful immunotherapeutics in association with different types of cancers (240). The summary of the different TLRs expressing the various immune cells across the TME is depicted in **Table 2**.

**Table 2:** Level of immune cell infiltration status mediated by the activation of TLRs in various cancers.

Cancer(s)	Associated TLRs	Level of expression	Type of TLR-producing immune cells in tumor cells	References
Head and neck cancer	TLR3	↑	NK cells, cDCs	(229)
	TLR4	↑	Neutrophils, cDCs, monocytes, eosinophils	
Esophageal squamous cell cancer	TLR3	↑	NK cells, cDCs	(229)
	TLR4	↑	Monocytes, cDCs, neutrophils, eosinophils	
	TLR7	↑	Monocytes, pDCs, eosinophils, neutrophils, B-cells	
	TLR9	↑	pDCs, monocytes, eosinophils, neutrophils, B-cells	
Lung cancer	TLR3	↑	NK cells, cDCs	(229)
	TLR4	↑	Monocytes, cDCs, neutrophils, eosinophils	
	TLR7	↑	Monocytes, pDCs, eosinophils, neutrophils, B-cells	
	TLR8	↑	Monocytes, cDCs, neutrophils, T regulatory (Treg) cells, T-cells	
	TLR9	↑	pDCs, monocytes, eosinophils, neutrophils, B-cells	
Breast cancer	TLR2	↑	cDCs, NK cells, monocytes, neutrophils, B- and T-cells	(229)
	TLR3	↑	NK cells, cDCs	
	TLR4	↑	Monocytes, cDCs, neutrophils, eosinophils	
	TLR5	↑	NK cells, cDCs, neutrophils, monocytes, T-cells	
	TLR9	↑	pDCs, monocytes, eosinophils, neutrophils, B-cells	
Pancreatic cancer	TLR4	↑	Monocytes, cDCs, neutrophils, eosinophils	(229)
	TLR7	↑	Monocytes, pDCs, eosinophils, neutrophils, B-cells	
Hepatocellular cancer	TLR2	↑	cDCs, NK cells, monocytes, neutrophils, B- and T-cells	(229, 241)
	TLR3	↑	NK cells, cDCs	
Gastric cancer	TLR2	↑	cDCs, NK cells, monocytes, neutrophils, B- and T-cells	(229)
	TLR3	↑	NK cells, cDCs	
	TLR4	↑	Monocytes, cDCs, neutrophils, eosinophils	
	TLR5	↑	NK cells, cDCs, neutrophils, monocytes, T-cells	
	TLR9	↑	pDCs, monocytes, eosinophils, neutrophils, B-cells	
Colorectal carcer	TLR3	↑	NK cells, cDCs	(55, 242)
	TLR4	↑	Monocytes, cDCs, neutrophils, eosinophils	
	TLR7	↑	Monocytes, pDCs, eosinophils, neutrophils, B-cells	
	TLR8	↑	Monocytes, cDCs, neutrophils, T regulatory (Treg) cells, T cells	
	TLR9	↑	pDCs, monocytes, eosinophils, neutrophils, B-cells	
	TLR10	↑	pDCs, monocytes, eosinophils, neutrophils, B-, T-, Treg cells	
Myeloma	TLR1	↑	Conventional DCs (cDCs), pDCs, monocytes, neutrophils, eosinophils, NK cells, B-cells	(229)
	TLR4	↑	Monocytes, cDCs, neutrophils, eosinophils	
	TLR7	↑	pDCs, eosinophils, monocytes, neutrophils, B-cells	
	TLR9	↑	pDCs, monocytes, eosinophils, neutrophils, B-cells	
Liver cancer	TLR4	↑	Monocytes, cDCs, neutrophils, eosinophils	(55, 229, 241)
	TLR9	↑	pDCs, monocytes, eosinophils, neutrophils, B-cells	
Ovarian cancer	TLR3	↑	NK cells, cDCs	(229)
	TLR4	↑	Eosinophils, neutrophils, cDCs, monocytes	
	TLR5	↑	NK cells, cDCs, neutrophils, monocytes, T-cells	
	TLR9	↑	pDCs, eosinophils, neutrophils, monocytes, B-cells	
Myelogenous leukemia	TLR2	↑	cDCs, NK cells, monocytes, neutrophils, B- and T-cells	(229)
	TLR3	↑	NK cells, cDCs	
	TLR4	↑	Eosinophils, neutrophils, cDCs, monocytes	
	TLR9	↑	pDCs, monocytes, eosinophils, neutrophils, B-cells	

The value of ↑ used in the table indicate upregulation/overexpression.

## 4 Sex-related TLRs (TLRs 7 and 8) in cancers

In accordance with sex chromosomal biology, men bear a paired chromosome of XY while the female gender possesses an XX chromosomal pair. Furthermore, in comparison with the Y chromosome the X chromosome bears a higher number of genes. This significant property of the X chromosome leads to the occurrence of two-folded copy numbers of the majority of X-dependent genes in women rather than men. Due to this knowledge, the overloaded number of X-linked genes is usually turned off to neutralize the imbalanced gene dosage in the cells of the female gender of mammals. This process is done through the activation of 1/2 of the female X chromosomes (243). In accordance with the reported results, in both mammals, humans, and mice, the *TLR7* and *TLR8* loci are located adjacent to each other. These genes are mapped to Xp22.3 and Xp22 (upon the short arm of the X chromosome), respectively. It seems that *TLR7* and *TLR8* genes are duplicated features rooted in a united ancestral gene (11, 54, 243, 244).

The capacity of the X chromosome is 155 Mb involving >1000 genes encoding miRNAs and proteins that contribute to immune responses. Recent reports reveal that in some cases e.g., B cells, monocytes, and plasmacytoid dendritic cells (pDCs) in women, *TLR7* does not comply with the X chromosome inactivation rule (243). According to the reported results, a limited portion (10%) of X-linked genes bear inactivation codes in the form of variable patterns. This heterogeneity leads to variation in X-dependent gene expression in females. In other words, in some women, both of the alleles (copies) will be expressed while in some others only one of the alleles will be expressed and the other alleles will be inactivated (245, 246). Moreover, TLR7 and TLR8 together with TLR3 and 9 bind to viral ligands. Interestingly, TLR7 and TLR8 are bi-zonal glycoproteins and are known as cell-surface and intracellular TLRs (11).

The X chromosome bears a versatile of important genes (e.g., CD40 ligand (*CD40L*), C-X-C Motif Chemokine Receptor 3 (*CXCR3*), forkhead box P3 (*FOXP3*), *TLR7* and *TLR8*) which apparently or hidden contribute to immune responses against microbial pathogens such as viruses (245, 247). As previous studies show, the highly conserved content of the mammalian X chromosome is an effective limitation for genetic exchanges between the X-gender chromosome and the autosomal ones. In this regard, X-linked inactivation is known as an effective regulatory feature to balance the likewise levels of X-linked genes both in male and female humans. Moreover, the upregulation of the X chromosome (about two folds) supports a determined balance between X chromosomal and autosomal gene expression. Thus the key genes that contribute to the immune system are not exchangeable between the X gender chromosome and the autosomal ones (245, 248). The inactivation of the X chromosome is known as an evolutionary mechanism for dosage equalization of gene expression in both gender of females and males; however, some genes including those that are involved in immune responses escape silencing. This feature may lead to an increase in the level of immunoprotein production in the female gender. Furthermore, the rate of mosaicism varies among female populations. These data explain why the level of expression of X-linked genes between females and males differ and even why the expression of these immune genes varies between female genders. In accordance with the reported results from previous investigations, the mosaicism feature in females is an advantage associated with deleterious mutations in X-linked genes and simultaneously provides a wide range of biological activities and immune responses among the mammalian female populations. Several X-linked genes involved in the immune system are recognized as genes with variable levels of expression in association with X chromosome inactivation. In this regard, important and key genes such as NF-κB activating protein (*NKAP*), *IRAK1*, and inhibitor of NF-κB kinase-γ (*IKBKG*) are recognized. As already discussed, the presence of clear variability in patterns of gene expression results in the induction of signaling pathways within the immune system which may lead to different immune responses both in female and male individuals (245, 246). According to the aforementioned characteristics of the X chromosome, any changes and alternations in DNA sequences and genes located on the X chromosome may lead to the occurrence of autoimmune diseases; therefore, ~80% of patients with autoimmune diseases belong to female individuals (249, 250).

### 4.1 Nucleic acid-sensing TLRs

Among 10 human TLRs (hTLRs), TLR3, 7, 8, 9, and 13 are recognized as nucleic acid (NA)-sensing (NAS) TLR glycoproteins. In this regard, a functional TLR3 is induced via a double-stranded RNA (dsRNA) while the TLR7 and TLR8 are activated through ssRNA fragments bearing determined and preferred sequences. In contrast to TLR3, 7, 8, and 9 as a NAS TLR is activated by single-stranded DNA-bearing motifs of unmethylated CpG. Thus, NAS TLRs are the body’s effective arsenal of weapons to identify viruses. All viruses encompass RNA or DNA molecules; the only detectable features by the human innate immune system are glycoproteins of NAS TLRs (10, 11, 251, 252). Although TLRs are useful weapons against strangers and pathogens, they sometimes are functional against the host’s self-NAs which results in a wide range of autoinflammatory disorders and autoimmune diseases e.g., systemic lupus erythematosus (SLE), rheumatoid arthritis, psoriasis, etc. (245, 251, 253).

The NAS TLRs with uncleaved ectodomains can bind to their related ligands. However, as per the previously reported results, the activation of NAS TLRs should be achieved through proteolytic cleavage processing. The proteolytic compartmentalization process of NAS TLRs ectodomains is performed by specific protease enzymes together with many others which may lead to the occurrence of effective responses by these immunoglycoproteins in the presence of their related ligands; because the proteolytic cleavage processing of NAS TLRs ectodomain provides a stable situation for TLR dimers and their activity in consequence (254–258). As reported results show, the proteolytic cleavage processing in NAS TLRs ectodomain structures is effectively done at acidic pH within the lysosomal and endosomal structures. Moreover, the trafficking process and the environment of the endoplasmic reticulum make NAS TLRs non-functional biomolecules. This condition decreases the induction of NAS TLRs activation against the host’s self-NAs. Among NAS TLRs, the ectodomain structures of TLR7 and TLR8 in the human body go through proteolytic cleavage processing in pH 7. This process is done via furin-like pro-protein convertase enzymes. Due to this knowledge, the activated structures of hTLR7 and hTLR8 occur out of the acidic environment of endosomes and/or lysosomes. Particularly, the hTLR7 and hTLR8 are early-activated NAS TLRs in human beings (245, 259, 260). The process of proteolytic cleavage in immunoglycoproteins of TLR7, 8, and 9 is performed at the Z-loop located on the extracellular ectodomain region between LRR14 and LRR15. The inactive form of TLR8 is dissimilar to TLR7 and 9 and the other hTLRS is in a dimeric form. The binding process of ligands to TLR8 active binding sites changes the spatial structure of inactive dimeric TLR8 into active dimeric TLR8 (261–263).

Up to now, four general mechanisms (e.g., internalization of NAs as ligands (through different mechanisms); NA digestion (NAs processing to generate ligands); NA digestion by nucleases such as DNA endonuclease of DNase I-like 3 (DNASE1L3) (as a negative regulator to decrease the NA ligands availability); physically ligand sequestration (out of the endosomal structures) are identified which mediates the ligand availability for NAS TLRs as an effective strategy against self- and non-self NAs (251).

In some cases, comprising macrophages (MΦs) and dendritic cells which are known as effective phagocytotic cells, the uptake of microorganisms is achieved easily; while in many other cells including B cells, the uptake of microorganisms and NAs (e.g., self-RNA and self-DNA) is associated with receptor-ligand interactions. In this regard, the TLR7 and 9 together with B cell receptors are involved in the synergistic activation of B cells associated with self-RNA and self-DNA forming immune complexes (251, 264, 265). In addition to the aforementioned cases, the antimicrobial peptide of LL37 which is also recognized as CAMP contributes to cellular uptake and complex formation constructed by self-RNA and self-DNA with LL37. This complex increases the endosomal uptake of condensed NAs and decreases the nuclease-related NAs degradation (As it happens when a protein like HMGB1 binds to self-NAs). In accordance with the reported results, the LL37, self-RNA, and self-DNA complexes induce the expression of IFN-I via TLR7 and 9 biomolecules in pDCs (251, 266–269).

As neutrophils are effective sources for the expression of LL37 at high levels and simultaneously are involved in extracellular traps. The LL37, self-RNA, and self-DNA complexes detect the neutrophil extracellular traps (NETs). The NETs are the probable main resource of self-NAs which are involved in psoriasis and SLE (267, 270, 271). Although the endosomal acidic environment leads to microbial lyses and the release of their NAs, some members of NAS TLRs including TLR8 are activated via nuclease-processed NAs. In this regard, it is presumed that RNase 2 and RNase T2 belonging to lysosomal endoribonucleases contribute to the recognition of TLR8-dependent RNA via cleaving the left nucleotides rather than uridine residues to generate specific TLR8 ligands obtained from some microbial RNAs pertaining to pathogenic microorganisms (272, 273).

The occurrence of mutations in DNASE1L3 results in SLE in children and autoimmunity development in mice that suffer from the lack of DNASE1L3. Both TLR7 as an RNA sensor and TLR9 are involved in this feature. The role of TLR7 as an RNA sensor may be associated with its ability to respond to deoxyguanosine or its contribution to a competition with the DNA sensor of TLR9 (274–280). TLR7 and 8 are activated by the short fragments of ssRNAs or single nucleosides. Thus, in the presence of these RNA biosensors application of nucleases to digest NAs into short fragments is not a logic solution to prevent the recognition of self-NAs (262, 263, 277, 281).

The NAS hTLRs similar to other hTLRs bear three sections in their structures including an intracellular or cytosolic domain of TIR, an extracellular domain of LRR, and a transmembrane helix. The process of ligand binding which is performed via the extracellular domain of TLR triggers TLR biomolecule to be dimerized and in consequence, the related downstream signaling pathways going to be activated. TLR7 can be activated by an ssRNA ligand, and it requires a single guanosine nucleoside molecule at the first binding site and a trimeric ssRNA with a uridine dimer at the second binding site to become activated. Therefore, similar to TLR8, TLR7 also contains two functional binding sites. In contrast to TLR7, TLR8 is activated by the presence of uridine within its first binding site and a dimeric molecule of uridine and guanosine within the TLR8 second binding site. These ligands have a synergistic effect on TLR8 activation (11, 251, 262, 263, 282).

In accordance with evolutional characteristics, TLR7 and 8 immunoglycoproteins have a high rate of homology in both their functional activities and structural sequences. Simultaneously, the first binding sites belonging to TLR7 and 8 are conserved and bind to a single nucleoside molecule; while the second binding site belonging to TLR7 has a synergistic effect on the function of the first binding site and has a different spatial structure rather than the second binding site in TLR8. However, the uridine of both TLR7 and 8 are also able to recognize imidazoquinoline compounds and guanosine analogs (e.g., 7-thia-8-oxoguanosine, 8-hydroxydeoxyguanosine, ioxoribine and deoxyguanosine) (11, 54, 262, 283). TLR7 and TLR8 bind to natural ligands e.g., hepatitis C virus ssRNA (284), HIV type-1 (HIV-1) ssRNA (285), and influenza A virus ssRNA (286).

## 5 TLRs as therapeutic targets for treating human cancers

As stated in the earlier sections, TLRs play a crucial role in maintaining immune homeostasis within the human body. An increase in the expression of TLRs or a positive modulation in the activation status of the TLRs leads to the hyperactivation of crucial transcription factors (NF-κB, AP-1, IRF-3) and secretion of several pro- and anti-inflammatory cytokines resulting in early innate responses and subsequent release of specific immune mediators. TLRs play a dual regulatory role in the TME by the induction of inflammatory responses within this area, which in turn contributes to immune cell infiltration, recruitment, and proliferation to escape the immune cells. On the other hand, the innate and adaptive responses elicited by the TLRs could regulate the anti-tumor activities. Recognition of activation mechanisms and computational simulation of molecular dynamics are efficient techniques to gain comprehensive insights into the structural and functional properties of proteins like TLRs, which in turn aid in designing effective drugs. Computational simulation of molecular dynamics represents a suitable illustration to understand the properties of interactions that can be occurred between receptors and their related ligands (262, 287–298). These significant data and information provide us with a promising opportunity to develop affordable, efficient, and straightforward therapeutic procedures in this field.

### 5.1 TLRs-binding molecules as therapeutic targets for treating human cancers

In the previous section, we have incorporated an overview of the roles of different TLRs in different human cancer subtypes. This highlights their possibility to play a critical regulatory role in chronic inflammation to induce immunosuppression and immune evasion of the tumor cells. Alternatively, TLRs can activate the M1 macrophages which drive antitumor responses through indirect antitumor activity, especially by direct cytotoxicity, antibody-dependent cell-mediated cytotoxicity (ADCC), and transactivating the CTLs (299). Overall, activation of TLR signaling presents a kind of balance between tumor-promoting and antitumor activity (300). Recognition of activation mechanisms and *in-silico* analyses are presently considered effective tools for an in-depth understanding of the structure-function relationship of TLRs to design effective natural/synthetic/semi-synthetic agonists or antagonist molecules (296). Many studies were focused on TLR agonists due to their easy clinical applications in cancer therapy. In fact, the adjuvant properties of TLR agonists in the activation of immune systems joint with the possibility to use them in combination with other anti-cancer treatments have shifted the attention to them instead of the antagonist molecules (300).

The response of the TLR-mediated and/or TLR-influenced signaling pathways has been found as the critical determinant in the success of TLR-based immunotherapy against cancers (229). However, the overt immune response in cancer and various autoimmune diseases directed the researchers to focus on several TLR antagonists for counteracting the overactivation of the TLR signaling cascade and thereby regulating the innate immunity (301). The TLR antagonists are mainly categorized into two different types, direct inhibitor and indirect inhibitor. Herein, the direct inhibitors are mainly the structural analogs of the TLR ligands/agonists and thereby compete with the TLR ligands to bind with the receptor and block the signaling cascade to alter the mode and intensity of the immune responses (302). While the indirect inhibitors are mainly the various anti-cancer agents that can hinder the activation of the TLR signaling pathways (302). Interestingly, a few published literatures are available regarding the investigation of the efficacy of TLR antagonists through preclinical/clinical trials for future use as therapeutic strategies against cancer. In the recent past, studies on several gut-related cancer types have documented the efficacy of a humanized anti- TLR2 antibody namely OPN-301 in suppressing the inflammation-driven tumorigenesis and proliferation of the gastric tumor cells in accordance with the downregulation of the expression of CXCL2 and TNF-α in murine model (303). Another small molecule inhibitor of TLR4 namely TAK-242 (Resatorvid) has been found to bind to the TIR domain of TLR4 to inhibit the downstream activation of the signaling molecules and transcription factors by hindering the interaction of TLR4 with TRAM and/or TIRAP (304). Studies have shown that TAK-242 have significant tumoricidal properties against breast cancer and ovarian carcinoma that majorly conferred through the regulation of the activation of NF-κB signaling pathways and expression of the p53-dependent apoptotic genes (305–307). Furthermore, this molecule can also reduce the enzymatic activity of MMPs and can block the transition of epithelial to mesenchyme for tumor formation in breast and ovarian tumor cells respectively (305–307). Interestingly, a naturally derived sesquiterpene lactone, Atractylenolide I (AO-1) has been screened for its excellent efficiency in inducing conformation change in TLR4-MD-2 complex resulting in the downregulation of the signaling molecules of MyD88-dependent NF-κB signaling axis (293). Downregulated NF-κB further contributes to reduce the secretion of IL-6, TGF-β1, VEGF, and IL-17A in tumor cell and increase the level of regulatory T cells that collectively inhibits the progression of epithelial ovarian cancer (308). Other TLR4 antagonists like paeonol (PAE) and CXC195 have shown promises as potential anti-cancer drugs that function through regulating the activation of the TLR4-MAPK/NF-κB pathway in human osteosarcoma as well as hepatocellular carcinoma as derived from their efficacy in cell culture experiments (309, 310). IMO-8400, an oligonucleotide-based antagonists of TLRs 7, 8, and 9 has also shown promising results in improving the severity of pathology of B-cell lymphoma types, especially resulted from L265P mutation in MyD88 (311, 312). This antagonist was found efficacious and safe in both phase I and II clinical trials conducted in the patients with different B-cell lymphoma types (312). However, Phase I trials with this compound on lymphoma patients (NCT02252146) and Waldenstrom’s macroglobulinemia (NCT02092909) were not satisfactory and hence terminated (303). Collectively, from the experimental and clinical trial data, TLR antagonists possess lower therapeutic potency in comparison to the agonists specifically treating the aggressive forms of various cancers. Ultimately, the function of these modulatory molecules for TLRs can be simplified to the concept that agonists serve as immunotherapeutic or vaccine adjuvants in the treatment of cancer, allergies, and infectious diseases, whereas antagonists hold potential as therapeutic approaches for addressing chronic inflammatory and autoimmune diseases (13).

#### 5.1.1 TLR1/TLR2

One of the first agonists discovered for the TLR1/TLR2 heterodimer was the triacylated lipopeptide Pam3CSK4 that allowed to initiate the signaling cascade (313). Successively, the small molecule named CU-T12-9 (N-methyl-4-nitro-2-(4-(4-(trifluoromethyl)phenyl)-1H-imidazol-1-yl)aniline) was developed through a high-throughput screening (181). This molecule shows a considerable overlap between its binding pocket and the binding site for the amide-bound lipid of Pam3CSK4 (314). CU-T12-9 can form stable hydrogen bonds with TLR1 and facilitates TLR1/2 heterodimer formation through hydrophobic interactions (13). A similar binding mode as CU-T12-9 was observed with CU-CPT22 (3,4,6-Trihydroxy-2-methoxy-5-oxo-5H-benzocycloheptene-8-carboxylic acid hexyl ester) molecule that acts as an antagonist able to compete with Pam3CSK4 for binding to heterodimeric TLR1/2 complex. In addition to inhibiting TLR1/2 signaling, CU-CPT22 exerts no effect on other TLRs (315). After screening a synthetic library of 14,000 compounds, the small molecule SMU-Z1 (2-(1-(2-(Methylamino)-5-nitrophenyl)-1H-imidazol-4-yl)-5-(trifluoromethyl)phenol) was recently discovered as one of the newest agonists (248). This molecule shows a strong antitumor immunity against leukemia and could be a potential drug candidate for TLR2-mediated antitumor immunotherapies (316). In anti-tumor applications, the main TLR2 agonists are the Bacillus Calmette–Guerin (BCG), the OM-174, and SMP105 molecules and their functions are described in **Table 3**.

**Table 3:** Agonists and antagonists of TLRs and their application as anticancer drugs.

Name of the TLR(s)	Agonist/Antagonist	Associated cancer	Mechanism of action	References
TLR1/ TLR2	MALP-2 (G)	Pancreatic cancer	Inhibits tumor growth and reverses tumor-associated immunosuppression	(317)
	Agonists: Polysaccharide-K (G)		Inhibits tumor growth and induces apoptosis in tumor cells	(318)
	*Bacillus* Calmette–Guerin (BCG)	Bladder cancer	Used in intravesical therapy; shows antitumor activity by induction of IFN-γ and IL-2. It increases the survival of the patients	(319)
	Pam3CSK4	Lymphoma	Reduces tumor load and increases the survival of the recipients	(13, 320)
TLR3	Polyriboinosinic-polyribocytidynic acid (Poly I:C)	Gastric cancer	Upregulates the pro-apoptotic genes and promotes apoptosis of the cancer cells	(35)
	Poly(A:U)	Breast cancer	Reduces the risk of recurrence and metastasis	(102)
TLR4	Monophosphoryl lipid A (MPLA)	Colorectal cancer	Acts as a vaccine adjuvant	(321)
	Brucella lumazine synthase (BLS)	Melanoma	Induces the secretion of INF-γ and increases the ratio of effector to regulatory cells	(36)
	Resatorvid (TAK-242)	Breast cell carcinoma and ovarian cancer	Regulate NF-κB signaling pathways and p53-dependent apoptotic genes, reduce the enzymatic activity of MMPs and epithelial to mesenchyme transition	(305–307)
TLR7	R-837 (Imiquimod or aldara)	Oral squamous cell carcinoma	Decreases cell growth around the transformed tissue and increases apoptotic cell death. It causes increased production of IFN-γ and less IL-10	(6)
		Breast cancer	Inhibits the growth of cutaneous breast cancer cells	(101)
		Melanoma (Preclinical phase)	Topical application of 5% imiquimod cream on primary melanoma results in an increase in the number of CD4+ and CD8+ T cells in the skin	(322, 323)
	R848 (resiquimod)	Cutaneous T-cell lymphoma (PhaseI)	Topical administration of resiquimod affects the early stage of cutaneous T-Cell lymphoma	(322, 324)
	852A	Breast-, Cervix-, and Ovarian cancers (Preclinical phase)	Enhances the expression of IP-10 and IL-13 (side effect: cardiac toxicity)	(322, 325)
		Lymphomatic leukemia (Phase I/II)	Induces the expression of IgM and inflammatory cytokines	(322, 326)
		Melanoma (Phase II)	Enhances the expression of IFN I and IP-10 within the serum	(322, 327)
	Loxoribine (guanine ribonucleotide derivative)	B-chronic leukemia (Preclinical)	Increases the fludarabine (a purine analog) activity which leads to the induction of apoptosis. Loxoribine together with fludarabine and mafosfamide exert a synergistic effect for apoptosis induction	(322, 328, 329)
TLR8	R848 (resiquimod)	Cutaneous T-cell lymphoma (PhaseI)	Topical application of resiquimod affects the early stage of cutaneous T-Cell lymphoma	(322, 324)
	VTX-2337	Lymphoma (Phase I)	Enhances the expression of G-CSF, macrophage inflammatory protein-1β, monocyte chemoattractant protein-1, and TNFα in the plasma	(330)
TLR9	CpG-ODN	T-cell lymphoma (Phase I)	PF-3512676 (formerly CPG 7909) as a TLR9 agonist exerts antitumor activity against refractory cutaneous T-cell lymphoma	(330, 331)
		Lymphoma (phase I/II)	Triggers tumor-reactive memory CD8 T cells that lead to systemic antitumor immunity	(330, 332)
		Neoplastic meningitis (Phase I)	Not determined	(330, 333)
	IMO	Non-small cell lung (Phase II)	IMO-2055 acts as a TLR9 agonist in combination with bevacizumab and erlotinib and is capable to increase the anti-tumoral activity via stimulation of the immune system	(330, 334)
	1018 ISS	Lymphoma (Phase II)	Enhances antigen expression, antibody-dependent cell-mediated cytotoxicity, and T helper cell type 1 shift	(330, 335)

#### 5.1.2 TLR3

Agonists of TLR3 have the potential to be effective candidates for antitumor therapies as they can activate the adaptive immune system response (336). A unique candidate, CU-CPT4a was detected between 1.2 million compounds in a different database. Before this, no approval was given to small-molecule TLR3-binding. Molecular docking analysis showed different binding points of CU-CPT4a with TLRs characterized by the presence of asparagine residues (337). Amongst the various agonist molecules used in anti-cancer therapy, the most known molecules are the Poly(I:C), Poly(I:C)12U, and poly-IC : LC which have shown encouraging results in inducing tumoricidal responses alone and in combination with radio- and chemo-therapy (338).

#### 5.1.3 TLR4

Since TLR4 is potentially the most efficacious PRR in regulating the growth and progression of various cancers, several TLR4-modulating molecules have been developed so far. In this context, TLR4 adjuvants are the primary contributors for developing various vaccine candidates (339). The US-FDA has already approved the use of BCG as an initiator for the TLR4 signaling pathway to generate anti-tumor immune responses against various types of cancers (340). BCG targets the binding of LPS to the hydrophobic pocket of MD2, preventing the dimerization of TLR4, and hindering the activation of the TLR4 signaling pathway (7). In this direction, bacterial derivatives such as *E. coli-*derived lipid A (OM-174) as potential therapeutic candidates are under the clinical trial phase for elucidating the TLR4-induced immune response against melanoma (6). Other TLR4 agonists like monophosphoryl lipid-A trigger the activation of TLR4 and are used as a therapeutic agent against lung carcinoma (341). TLR4 initiates an intracellular signaling cascade and activates cytoplasmic signaling pathways, ultimately resulting in the expression of PDL-1 (programmed death ligand-1). This creates an anti-inflammatory response that helps to cease the metastasis. In addition, the expression of TLR4 can influence the proliferation patterns in cancer stem cells and glioblastoma multiforme cells (342).

#### 5.1.4 TLR5

Flagellin, a TLR5 agonist, is widely used in anti-cancer treatment. Studies by Sfondrini et al. (343) have shown that the generation of TLR5-flagellin nanoparticles imparts anti-tumor function in an experimental animal model of cancer. In breast cancer, the TLR5 agonist activates the intrinsic signaling pathway leading to the upregulation of IFN-γ and IL-4 population and lowering of the proportion of regulatory T cells that results in cell proliferation and suppressing the tumor growth (343).

#### 5.1.5 TLR7/8/9

Agonists designed against the intracellular TLRs viz., TLR7, 8, and 9 play an important role in modulating the signaling pathways involved in the development of cancer. Among all the intracellular TLRs, TLR7/8 has the highest immunomodulatory effects. They are activated concurrently through the binding of ligands recognizing the ssRNAs. The most important and well-evident agonists of TLR7/8 include the guanine analogs like imiquimod. It has the potential for restoring the functional efficiency of effector T cells, and NK cells in the therapy against non-melanoma skin cancer. US-FDA and European Medicine Agency (EMA) have already approved the application of the imiquimod-based cream Aldara for the treatment of basal cell carcinoma. A study by Liang et al. (344) demonstrated the development of a novel adjuvant using the neoantigen peptides that are released form the colon cancer cell line for cancer immunotherapy. These peptides were charged with the consecutive application of positively charged lysine to generate a cationic polypeptide that shows affinity with TLR9 agonist CpG oligodeoxynucleotides (202).

In the aforementioned sections, we have discussed about the efficacy of different classes of natural/synthetic compounds as agonists and/or antagonists of TLRs in the context of developing anti-cancer therapeutics. Most of the compounds/peptides showing adequate efficacy in cell line and animal models have been tested for their therapeutic promise in humans through different phases of clinical trials. We have noted that the TLR agonists exhibit better efficacy and safety as an anti-cancer agent as compared to the antagonists. Taking this into the consideration, the current status of various TLR agonists active and/or recruited in the different clinical trials are listed in **Table 4**. These trials suggest that these compounds/peptides can effectively induce the expression of pro-inflammatory cytokines and stimulate the influx of effector T cells into or around the tumor(s) by activating the TLRs-mediated signaling circuit transforming “cold tumors” into “hot tumors”. However, in terms of acceptability and safety of application, several TLR agonists decipher a number of side-effects like flu-like symptoms, allergic reaction, fatigue, and decreased blood counts (345).

**Table 4:** Status of clinical trials with TLR agonists for therapeutic intervention of human cancers.

Targeting TLR	Name of Agonist/Drug	Name of Cancer	Intervention application	Mechanism of action	Clinical trial number
Active/recruited in PHASE I trial					
TLR3	Poly ICLC: Hiltonol	Breast carcinoma	In combination with vaccine, and chemotherapy	Intramuscular application is active; endogenous type I IFN inducer.	NCT02826434
					NCT03362060
		Glioma	In combination with vaccine (peptide) and surgery	Subcutaneous application is active; Induction of CD8+ T-cell responses.	NCT02960230;
					NCT02924038
					NCT02549833
		Prostrate cancer	Combination with surgery	Intramuscular recruiting trial; increases IFN-β and IFN-α expression in the circulating PBMCs.	NCT03262103
		Multiple myeloma	Combination with vaccine and chemotherapy	Recruited in the trial; high degree of T cell response.	NCT02886065
TLR 7	Imiquimod: R837, Aldara, UGN-201	Bladder cancer	Combination with surgery and drugs	Intravesical recruiting trial	NCT05375903
					NCT05055050
		Squamous cell carcinoma	Combination with chemotherapy	Topical recruiting trial	NCT03370406
		Solid tumors	Combination with vaccine and drugs	Topical recruiting trial	NCT04116320
					NCT03872947
		Oral cancer	Single-use drug	Topical, recruiting trial	NCT04883645
TLR7/8	BDB001	Solid tumors	Single-use in combination with drug	Recruited in the trial.	NCT03486301
					NCT04196530
TLR9	SD-101	Solids tumors	Combination with BMS 986178	Intratumoral application is active.	NCT03831295
		Lymphoma	Combination with radiotherapy and	Intratumoral application is active.	NCT03410901
		Pancreatic adenocarcinoma	Combination with Nivolumab and radiation	Intratumoral application is active.	NCT04050085
	CMP-001:Vidutolimod, ARB- 1598; CMP-001: CYT-003	Colorectal cancer	Combination with Nivolumab, Ipilimumab and Radiotherapy	Subcutaneous and/or intratumoral application is active.	NCT03507699
Completed PHASE I trial					
TLR3	Poly ICLC: Hiltonol	Acute myeloid leukemia, myelodysplastic syndrome	In combination with vaccine and chemotherapy	Induces expression of tumor antigen and induces cytotoxic T cells.	NCT01834248
					NCT03358719
		Solid tumors	Vaccine adjuvants; chemotherapeutic drug combinations	Elicits innate and humoral immune responses; increases tumor-infiltrating T cells.	NCT02721043
					NCT02897765
					NCT02544880
		Pancreatic cancer	vaccine	Elevates the intensity and duration of the immune responses.	NCT01677962
		Lungs cancer	vaccine	Activation of anti-tumor immune responses.	NCT03380871
		Melanoma	vaccine	Increased T cell response in synergy with vaccine candidate.	NCT01585350
TLR4	LPS	Melanoma	vaccine	Induces immune-responses and immunogenicity.	NCT01585350
	GLA-SE: G100	Merkel cell carcinoma	In combination with Surgery and radiotherapy	Less toxicity and efficient clinical acceptability.	NCT02035657
		Melanoma	vaccine	Effective vaccine adjuvant and increased immunopotency of vaccine.	NCT01585350
		Sarcoma	In combination with Radiotherapy	Ameliorates local sarcoma and induces proliferation of CD4+ T cell response.	NCT02180698
	GSK1795091 (CRX-601)	Solid tumors	Intravenous administration with immunoadjuvants	Inhibition of tumor growth with no adverse side effects.	NCT02798978
TLR5	entolimod	solid tumors	Intramuscular/subcutaneous single use	Reduces local tumorogenic responses.	NCT01527136
TLR7	Imiquimod	gastric cancer and breast cancer	In combination with Vaccine and chemotherapy	Topical application elevates anti-cancer immune responses.	NCT02276300
		Glioma	In combination with Vaccine	Stimulate immune responses by increasing the proliferation of IL-17 and IDH1 specific T cells.	NCT02454634
		Prostrate cancer	Vaccine	Induces strong immunogenic response.	NCT02293707
TLR7/8	Resiquimod: R848; S28463	Melanoma	Vaccine	Elicits humoral and CD4+ T cell responses.	NCT00821652
TLR8	Motolimod: VTX-378, VTX- 2337	Ovarian tumors	In combination with chemotherapy	Disease stabilization and amelioration of tumor.	NCT01294293
TLR9	CMP-001: Vidutolimod	Lungs carcinoma	In combination with Radiotherapy	Reduces disease burden.	NCT03438318
Active and/or recruiting PHASE II trial					
TLR3	Poly ICLC: Hiltonol	Brain tumors	Combination with vaccine	Active in trial	NCT01204684
		Melanoma	Intradermal and/or subcutaneous administration as vaccine adjuvant and antibody treatment	Active in trial	NCT02126579
					NCT04364230
	BO-112	Melanoma	In combination with drugs	Intratumoral application is active	NCT04570332
TLR7	Imiquimod (R837, Aldara, UGN-201)	Cervical intraepithelial neoplasia	Single use with vaccine	Recruited in the trial	NCT02864147
		Basal cell carcinoma	Combination with surgery and drugs	Recruited in the trial	NCT03534947
	Resiquimod (R848; S28463)	Brain tumor	Use with vaccine	Active in trial	NCT01204684
		Melanoma	Adjuvant for vaccine	Active in trial	NCT02126579
TLR9	SD-101	Prostrate carcinoma	Used with radiation	Recruited in the trial	NCT03007732
	Tilsotolimod (IMO-2125)	Solid carcinomas	Application with drugs	Active in trial	NCT03865082
		Malignant melanoma	Single application	Recruited in the trial	NCT04126876
	CMP-001 (Vidutolimod, ARB-1598, CYT-003)	Melanoma	Combination with surgery and drugs	Recruited in the trial	NCT04401995
					NCT04708418
					NCT04698187
					NCT03618641
		Breast cancer (triple negative)	Combination with radiotherapy	Recruiting trials	NCT04807192
Completed PHASE II trial					
TLR3	Poly ICLC (Hiltonol)	Melanoma	Single application as intratumoral and/or intramuscular	Induce systemic immune response.	NCT02873819
					NCT01079741
		Solid tumors	Combination with vaccine and drugs	Induction of tumor-specific immune response.	NCT02873819
					NCT02643303
	Rintatolimod (Ampligen, Atvogen)	Metastatic colorectal cancer	Used in chemokine modulation	Elicits anti-tumorigenic response.	NCT03403634
TLR4	IDC-G305	Multiple cancers: melanoma, renal-cell carcinoma, ovarian cancer	Single intramuscular dose	Efficacious therapeutic potential to generate antigen-specific immune response.	NCT02015416
TLR7	Imiquimod (R837, Aldara)	Stage 2 and 3 Cervical intraepithelial neoplasia	In application with surgery	Endorses the regression of cervical high-grade intraepithelial lesions.	NCT03233412
					NCT02130323
		Breast cancer	In combination with radiation and chemotherapy	Reduces disease burden	NCT01421017
					NCT00899574
		Prostrate cancer	Used as vaccine and incomplete Freund’s adjuvant	Triggers high immunogenic response.	NCT02293707
TLR9	SD-101	Solid tumors and lymphoma	applied with drugs and radiotherapy	Enhances antigen-specific immunity.	NCT02254772
					NCT03322384
	CpG7909	Mantle cell lymphoma, recurrent lymphoma	In combination with a vaccine, several drugs, and chemotherapy	Induce anti-tumor CD8 T cell immune response to reduce the clinical outcomes.	NCT00490529
Multiple TLRs: TLR2, 4 and 9	Bacille CalmetteGuérin (BCG)	Bladder cancer	Applied with vaccine and drugs	Intravesical application triggers anti-tumor immune response	NCT01373294
					NCT02015104
					NCT02792192

## 6 Advantages and limitations

Being TLRs the crucial components of innate immunity as well as adaptive immunity, TLR-based therapeutic approaches have both positive and negative impacts. Selective targeting of different TLRs with small molecules, chemical compounds, nanomaterials, peptides, and antibodies can satisfactorily inhibit TLR-driven inflammatory processes that are generally associated with cancer-associated inflammation and metastasis (35, 114, 317). At the same time, targeted stimulation of TLR activity can give rise to sensitized tumor-associated macrophages and cytotoxic T cells that kill cancer cells. However, the dose and duration of the treatment are the two most important criteria for obtaining beneficial outcomes. As the perturbation of TLR activity results in immune homeostasis disruption, this could be detrimental for the recipient. Nowadays, phytochemical-derived or natural products-inspired pharmacophores are employed in clinical trials. These molecules can fine-tune TLR activity in humans and restrict the growth of tumors without hampering the immune homeostasis of the recipient (346). Also, TLR polymorphism is one of the crucial genetic components in regulating the outcomes of different cancers. However, most of the studies reported so far were conducted with a limited number of participants. Therefore, to get meaningful conclusions about the influence of different TLR polymorphisms a higher number of study participants/subjects should be involved.

## 7 Future perspective and conclusion

**Figure 2:**
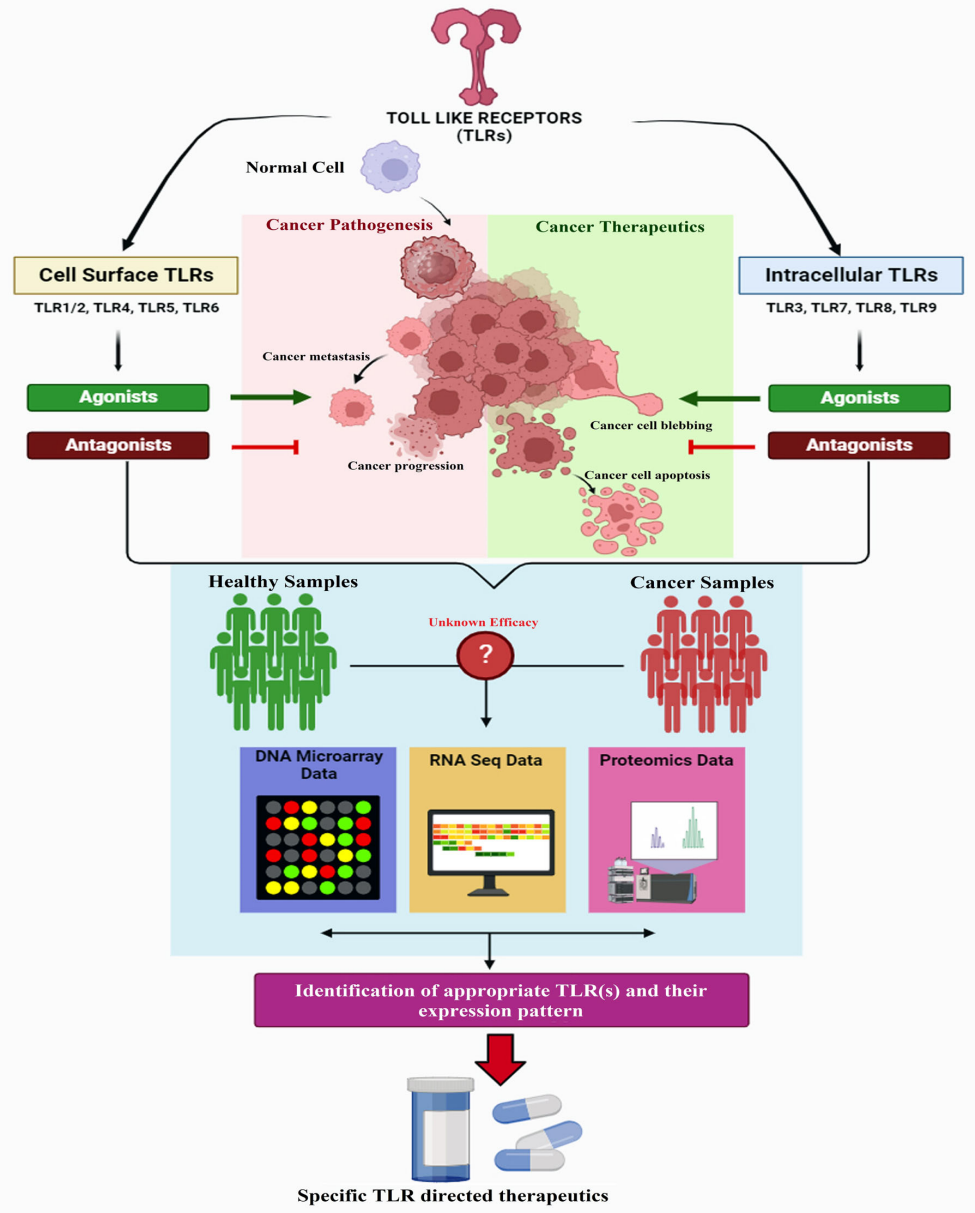
Scheme depicting the possible routes of chemotherapeutic/immunotherapeutic intervention of cancers through selection of appropriate TLR(s) and their pattern of expression.

TLRs are crucial components in the process of immunomodulation and immunotherapeutics against different cancers. In this review, we have comprehensively presented the overall role of TLRs in the pathogenesis of different cancers and their role in the development of anti-cancer therapy. As crucial host receptors and immune sensors in the innate immune system, targeting the activity of TLRs through chemo- and immunotherapies can be a promising approach for treating the pathological manifestations of various cancers, especially the inflammatory pathologies associated with metastasis and angiogenesis. Uncovering new polymorphic variants of TLRs and their association with increasing the risk of cancer and/or conferring protection against the same has been a pathbreaking achievement in recent times. Given the highly interconnected nature of TLRs and their signaling pathways, as well as their association with numerous physiological functions within the human body, it is crucial to adopt a precise targeting approach, otherwise, the treatment may cause fatal outcomes. In this direction, our study explores the yin and yang of TLRs in the progression of cancer to therapeutic intervention. In addition, the study of TLR networks of interaction could enhance our comprehension of TLR’s intimate functions. Activation of the TLR by administrating various agonists resulting in the downstream signaling cascade can potentially covert the “cold tumors” into “hot tumors” that can promote activities of various immune checkpoint inhibitors to mitigate the tumor (345). Several novel TLR agonists are being investigated as monotherapy, chemotherapy, and vaccine adjuvants in different patients with a range of cancers due to their potential to induce direct anti-tumor responses as well as to increase the clinical efficacy of the existing immunotherapeutic drugs. However, there are only a few publications and trials on the role of TLR antagonists as anti-cancer agents are available in the public domain. However, based on the available data, TLR agonists are found to be more efficacious than that of antagonists. Furthermore, the use of advanced tools (e.g., caging strategies, molecular dynamics, appropriate gene expression tools, and multiomics analyses at the genomics, transcriptome, and proteome levels), can provide further insight into the dynamics of TLR signaling. This comprehensive approach could significantly advance our understanding of the role of TLRs in diseases and their potential as prognostic markers (347–349). This review article is therefore expected to provide a wealth of information to the scientific communities to improve the understanding of the use of TLR-directed therapeutic strategy in combating different human cancers and to promote this strategy for field application to save the lives of unfortunate patients. A scheme on the possible approach for TLR directed chemo- and immunotherapy has been given as **Figure 2**.

## Author contributions

SM and PB conceived and planned the study and organized the manuscript. SM, RP, PB, AP, and MS performed a literature search and prepared the first draft of the manuscript. SM, RP, AP, and MS illustrated the picture. SM, PB, AP, AM, and MS provided expert input, discussed the conception of the review, and contributed to the final manuscript edition and preparation. SM, PB, and MS supervised the manuscript. All authors contributed to the article and approved the submitted version.

## Glossary

**Table:**

ALL	Acute lymphoblastic leukemia
B-ALL	Acute lymphoblastic leukemia
ADC	Adenocarcinoma
ATP	Adenosine triphosphate
ADV	Adenovirus
ADCC	Antibody-dependent cell-mediated cytotoxicity
AO-1	Atractylenolide I
BCG	Bacillus Calmette–Guerin
BE	Barrett’s esophagus
B-CLL	B-cell chronic lymphocytic leukemia
BCPALL	B-cell precursor acute lymphoblastic leukemia
BBMC	Bone marrow mononuclear cells
BLS	Brucella lumazine synthase
BL	Burkitt lymphoma
cagPAI	cag pathogenicity island
CSC	Cancer stem cells
CAF	Cancer-associated fibroblasts
CD40L	CD40 ligand
CLL	Chronic lymphocytic leukemia
CRC	Colorectal cancers
CXCR3	C-X-C Motif Chemokine Receptor 3
COX-2	Cyclo-oxygenase 2
DAMPs	Damage/danger-associated molecular patterns
DNMT2	DNA methyltransferase 2
dsRNA	Double-stranded RNA
EGF	Epidermal growth factor
EAC	Esophageal adenocarcinoma
ESCC	Esophageal squamous cell carcinomas
EMA	European Medicine Agency
FGF2	Fibroblast growth factor 2
FOXP3	Forkhead box P3
GC	Gastric cancer
GB	Glioblastoma
GSCs	Glioma stem cells
HBV	Hepatitis B virus
HCV	Hepatitis C virus
HCC	Hepatocellular carcinoma
HL	Hodgkin’s lymphoma
HIV	Human immunodeficiency virus
HPV	Human papillomavirus
hTLRs	Human TLRs
IKBKG	Inhibitor of NF-κB kinase-γ
IRF-3	Interferon regulatory factor-3
IL-1β	Interleukin
IARC	International Agency for Research on Cancer
LRR	Leucine-rich repeats
LPS	Lipopolysaccharide
MCL	Mantle cell lymphoma
MMP9	Matrix-metalloproteinase 9
MAMPs	Microbial/microbe-associated molecular patterns
miRNAs	MicroRNAs
MAPKs	Mitogen-activated protein kinase
MPLA	Monophosphoryl lipid A
MM	Multiple myeloma
MAL	MyD88 adapter-like
MyD88	Myeloid differentiation factor 88
MDSC	Myeloid-derived suppressor cells
NKAP	NF-κB activating protein
NLPHL	Nodular lymphocyte predominant form
NAFLD	Non-alcoholic fatty liver disease
NHL	Non-Hodgkin’s lymphoma
NSCLC	Non-small cell lung cancer
NF-κB	Nuclear factor-κB
NA	Nucleic acid
NAS	Nucleic acid-sensing
PAE	Paeonol
PDAC	Pancreatic ductal adenocarcinoma
PAMPs	Pathogen-associated molecular patterns
PRR	Pattern recognizing receptors
PBMC	Peripheral blood mononuclear cells
pDCs	Plasmacytoid dendritic cells
Poly I:C	Polyriboinosinic-polyribocytidynic acid
ROS	Reactive oxygen species
T reg cells	Regulatory T
SLE	Systemic lupus erythematosus
TH1	T helper 1
TTFT	Time to first treatment
TIRAP	TIR domain-containing adapter protein
TRIF	TIR-domain-containing adapter-inducing interferon-β
TLRs	Toll like receptors
TGF-β	Transforming growth factor-β
TRAM	TRIF-related adaptor molecule
TME	Tumor microenvironment
TNF-α	Tumor necrosis factor-α
MT1-MMP	Type 1 matrix-bound metalloproteinase
VEGF	Vascular endothelial growth factor
WHO	World Health Organization
XAMPs	Xenobiotic-associated molecular patterns
